# Comparative Molecular Dynamics Study of the Thermal Stability of CheY Proteins from Hyperthermophilic and Mesophilic Organisms

**DOI:** 10.1021/acs.jcim.5c02944

**Published:** 2026-03-10

**Authors:** Salomón J. Alas-Guardado, Melisa S. Anzures-Mendoza, José Y. Sol-Fragoso, Edgar López-Pérez

**Affiliations:** † Departamento de Ciencias Naturales, 103801 Universidad Autónoma Metropolitana Unidad Cuajimalpa, Ciudad de México 05348, México; ‡ Departamento de Química, Universidad Autónoma Metropolitana Unidad Iztapalapa, Ciudad de México 09310, México; § Department of Molecular & Cell Biology, 7712 University of Connecticut, Storrs, Connecticut 06269, United States

## Abstract

The primary function of the CheY protein is to regulate flagellar motility in motile bacteria such as *Escherichia coli* and *Thermotoga maritima*. Although the general determinants of thermal stability in CheY from the hyperthermophilic bacterium *T. maritima* (TmY) have been proposed, the molecular mechanisms that enable this protein to remain structurally and functionally competent at elevated temperatures are not fully understood. Here, we investigated the thermal stability of TmY through all-atom molecular dynamics simulations, using three independent trajectories of 1 μs each at five different temperatures. Equivalent simulations were performed for its mesophilic homologue from *E. coli* (EcY) to enable a direct comparison under identical conditions. Our observations show that TmY preserves its native fold and global compactness across the entire temperature range, whereas EcY exhibits progressive destabilization and unfolds at high temperatures. Mechanistically, the enhanced thermal resistance of TmY is associated with an extensive network of salt bridges that interconnect secondary-structure elements and couple the N- and C-terminal domains. These electrostatic networks act as stabilizing scaffolds that restrain local flexibility, preserve domain communication, and maintain a tightly packed globular architecture under thermal stress, providing a molecular basis for the superior stability of TmY relative to its mesophilic counterpart.

## Graphical abstract

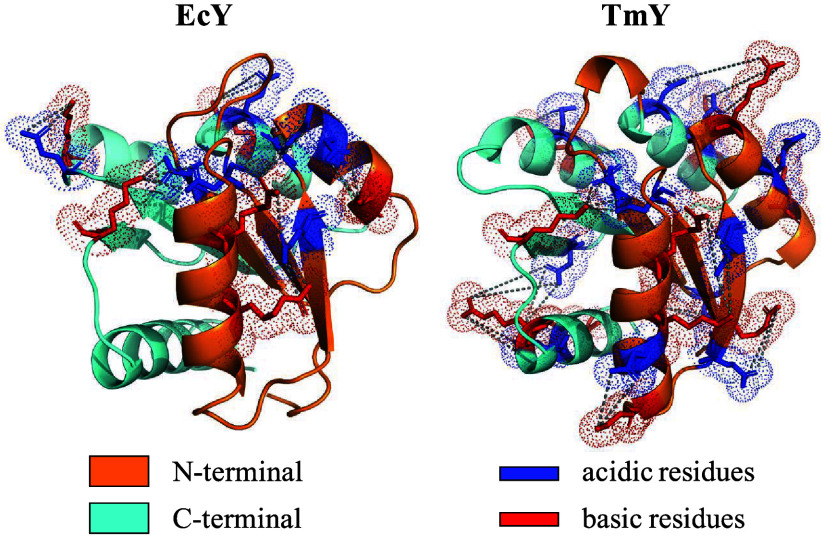

## 1 Introduction

For millions of years, living organisms have colonized a wide range of environments across the Earth, from temperate ecosystems to extreme habitats. Among them, bacteria are particularly notable due to their abundance and remarkable ability to thrive under diverse and often harsh conditions, including extreme temperatures.^1–3^ Based on their optimal growth temperature ranges, microorganisms are commonly classified as mesophiles, thermophiles, hyperthermophiles, and psychrophiles.^2,3^ Mesophilic organisms typically grow between 20 and 45 °C.^2^ Thermophiles grow optimally between 60 and 80 °C, whereas hyperthermophiles can survive at temperatures exceeding 80 °C.^2–6^ In contrast, psychrophiles are adapted to low-temperature environments, often growing at temperatures below 5 °C.^2^ This broad thermal adaptability has made microorganisms an important model for investigating the molecular and structural mechanisms underlying protein thermal stability.

Beyond thermal adaptation, the ability of microorganisms to thrive across diverse environments also relies on finely tuning cellular functions that depend on the structural stability and dynamics of their proteins. One such essential function is motility, which allows bacteria to efficiently respond to environmental changes. Bacterial motility is driven by rotary motor–like protein complexes that enable flagellar movement and directional control.^7,8^ Within this system, the CheY protein plays a key role as a signal transduction component of the chemotaxis pathway.^9–11^ Specifically, CheY regulates flagellar rotation in response to chemical gradients, as its phosphorylated state promotes clockwise rotation of the flagellum, thereby modulating bacterial swimming behavior.^12^

Understanding how proteins preserve their structure and function under extreme thermal conditions remains a central challenge in studies of thermophilic and hyperthermophilic organisms.^13–15^ Previous work has identified several structural and physicochemical features associated with enhanced thermal stability, including an increased number of salt bridges and hydrogen bonds, the formation of disulfide bonds, strengthened hydrophobic interactions, improved packing density, reduced internal cavities, and specific amino acid substitutions that promote structural rigidity.^14–16^ However, how these stabilizing features are distributed and coordinated within functionally critical proteins, particularly those involved in signal transduction and motility, remains insufficiently characterized.

In this context, the CheY protein from hyperthermophilic bacterium *Thermotoga maritima* provides an ideal model for probing the molecular basis of protein thermal stability. Although CheY is highly conserved in structure and function across motile bacterial species,^7,10^ its melting temperature is substantially higher in *T. maritima* than in mesophilic homologues such as those from *Bacillus subtilis* and *Escherichia coli*, with differences of approximately 46 and 35 °C, respectively.^11,16^ This marked thermal divergence among structurally similar proteins offers a unique opportunity to dissect the molecular determinants underlying the enhanced thermostability.

Computational approaches, particularly all-atom molecular dynamics (MD) simulations, have become indispensable for investigating protein dynamics and stability as biological function is closely linked to structural flexibility and atomic-level motions. MD simulations allow the systematic exploration of molecular interactions and conformational fluctuations under thermal conditions that are often challenging to access experimentally,^17–19^ making them especially well-suited for investigating the thermal stability of proteins.^20,21^

Therefore, in this study, we employed all-atom MD simulations to compare the CheY proteins from the hyperthermophilic bacterium *T. maritima* and the mesophilic counterpart *E. coli* at five different temperatures (302, 328, 374, 400, and 450 K). By analyzing key global and local structural and molecular descriptors, such as the root-mean-square deviation, radius of gyration, fraction of native contacts, root-mean-square fluctuation, secondary structure content, hydrogen bonding, and salt-bridge networks. We sought to elucidate the molecular mechanisms that enable hyperthermophilic CheY to maintain its structural integrity and biological functionality under extreme thermal conditions.

## 2 Materials and Methods

### 2.1 Molecular Models

Structural information for both CheY protein variants was retrieved from the Protein Data Bank (PDB, https://www.rcsb.org). The CheY protein from the hyperthermophilic bacterium *T. maritima* (PDB ID: 1TMY), hereafter referred to as TmY, has a molecular weight of 13.23 kDa and consists of a single polypeptide chain of 120 amino acids. The homologous protein from the mesophilic bacterium *E. coli* (PDB ID: 3CHY), hereafter termed EcY, has a molecular weight of 14.27 kDa and is composed of 128 amino acids, excluding the initial methionine residue.

Figure 1 shows the overall structural similarity between the CheY proteins (TmY and EcY). Sequence alignment reveals that the two proteins share 30% identical and 56% similar residues (Figure 1c). Despite this moderate sequence conservation, their secondary structure organization is largely preserved, as both adopt a compact, globular α/β fold consisting of five parallel β-strands forming a central β-sheet surrounded by five α-helices (Figure 1a,b).^9,10^ The main structural difference is located in the region between the β_3_-strand and the α_3_-helix, known as the “γ-turn loop”. In this region, TmY contains a short α-helix comprising three residues, whereas EcY presents a flexible loop.^9–11^ Using bioinformatic tools, specifically PyMOL v2.5.2 (https://pymol.org/) and the ESPript 3.2 server (https://espript.ibcp.fr/ESPript/ESPript/), this structural element was identified as 3_10_-helix and η1, respectively (Figure 1a,c). Additionally, the N-terminal domain comprises β_1_, α_1_, β_2_, α_2_, and β_3_, whereas the C-terminal domain includes α_3_, β_4_, α_4_, β_5_, and α_5_ in both proteins. The 3_10_-helix in TmY is located within the N-terminal domain.^22^

**Figure 1:**
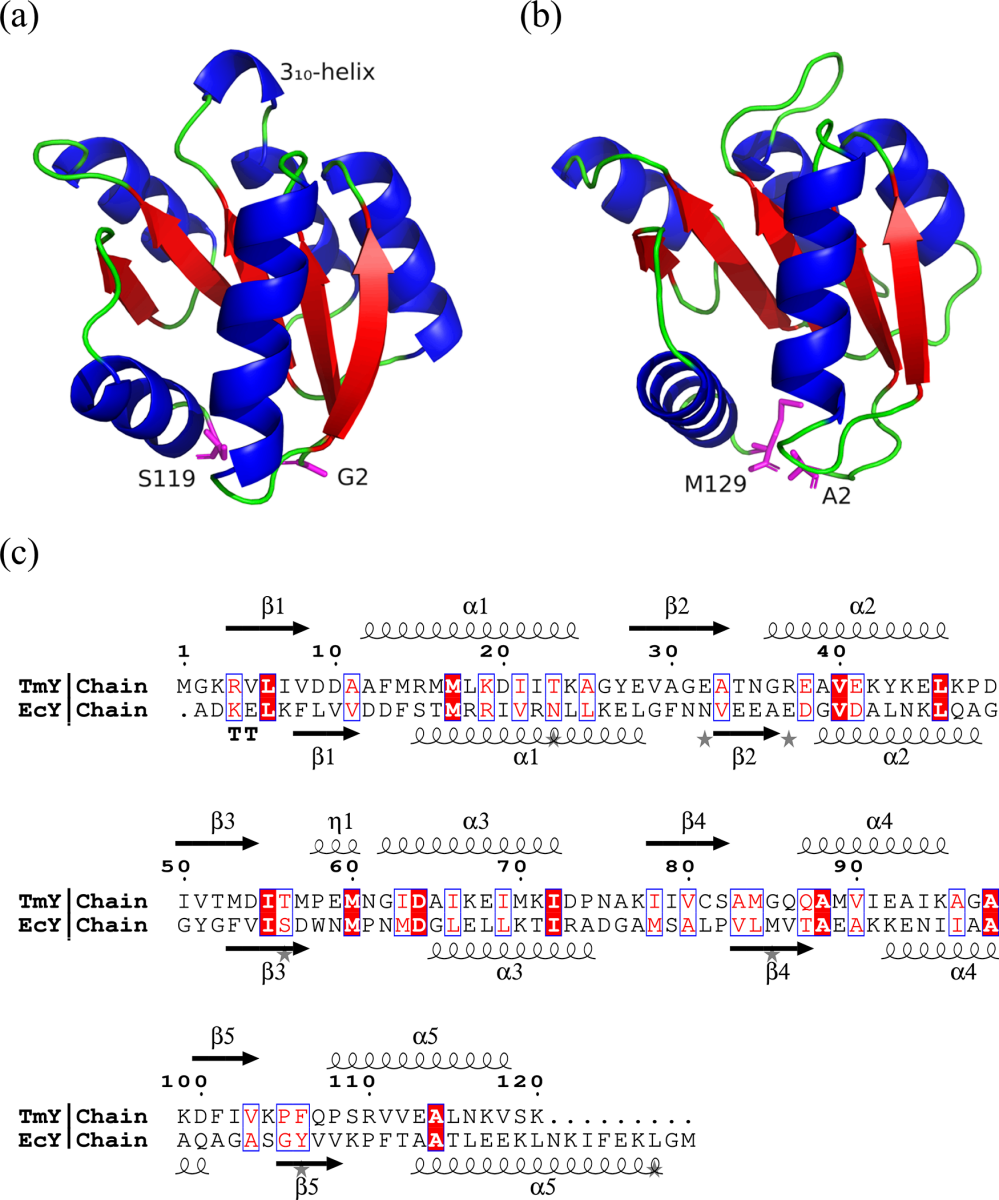
Cartoon representations of the CheY protein structures obtained from crystallographic data: (a) TmY and (b) EcY. The N- and C-terminal residues are highlighted in magenta. Note that the X-ray structure of TmY contains 118 residues, as the methionine (M1) and lysine (K120) residues are not resolved in the crystal structure. (c) Sequence alignment of both proteins generated from their FASTA files: identical amino acids are highlighted in red, and similar amino acids are marked in blue boxes and red type.

### 2.2 Molecular Dynamics Simulations

Since the crystallographic structures obtained from the PDB lack hydrogen atom coordinates, it was necessary to assign the protonation states of all titratable residues. The Adaptive Poisson–Boltzmann Solver (APBS) software coupled with the PropKa tool was used for this purpose at neutral pH (pH 7.0).^23^ After protonation, MD simulations were performed using the GROMACS package (v2020.3 and v2024.1)^24,25^ with the AMBER99SB force field.^26^ Prior to the production MD runs, the system was prepared by following the steps described below.

(a) The solvation boxes were first generated using a dodecahedral geometry, with the proteins centered at minimum distances of *L* = 1.0 nm and *L* = 2.0 nm from the box edges. This configuration ensured optimal periodic boundary conditions and prevented self-interaction between periodic images. The *editconf* module in GROMACS was used for this purpose. For the TmY protein, only *L* = 1.0 nm was employed, whereas for EcY, both distances were tested: *L* = 2.0 nm at 450 K and *L* = 1.0 nm for the remaining temperatures. The rationale for this parameter selection is described later.
(b) Each system was then solvated using the explicit SPC/E water model with the *solvate* module of GROMACS.
(c) Finally, the solvated systems were neutralized at pH 7.0 using the *genion* module of GROMACS, which replaces water molecules with counterions.

The TmY and EcY proteins comprise 1855 and 1981 atoms, respectively. For systems built with a distance of *L* = 1.0 nm, the number of solvent atoms was 14,379 for TmY and 14,898 for EcY, with the latter also containing four Na^+^ counterions for charge neutralization. Consequently, the total number of particles in the thermophilic and mesophilic systems was 16,234 and 16,871, respectively. When the distance increased to *L* = 2.0 nm, the EcY system contained 36,906 solvent atoms, resulting in a total of 38,891 particles.

Prior to equilibration, each system underwent an energy minimization step to remove unfavorable geometric contacts and steric clashes among particles, thereby relaxing local structural strains introduced during system preparation, including solvation and ion placement.^27^ This step ensures a physically reasonable initial configuration and prevents numerical instabilities during subsequent simulations. Energy minimization was performed using the *steepest descent* algorithm and converged after 667 and 321 steps, yielding potential energies (*E*_P_) of −2.60 × 10^5^ and −2.65 × 10^5^ kJ·mol^–1^ for the TmY and EcY systems at *L* = 1.0 nm, respectively. For the EcY system with *L* = 2.0 nm, minimization converged after 1047 steps, reaching *E*_P_ = −6.67 × 10^5^ kJ·mol^–1^.

The systems were then equilibrated sequentially using the NVT and NPT ensembles. Initial equilibration under the NVT ensemble was carried out to stabilize the system temperature at the target value while maintaining a fixed volume, thereby avoiding abrupt density changes. Subsequently, NPT equilibration allowed relaxation of the pressure and solvent density, ensuring proper system dimensions and solvent packing prior to the production MD runs. The equilibration protocol consisted of the following steps:

(a) NVT equilibration. Temperature equilibration was performed under the canonical (NVT) ensemble for 100 ps using an extension of the Berendsen thermostat, known as the *velocity-rescaling* method.^28^ The temperature was maintained at the target value for each simulation condition: 302, 328, 374, 400, and 450 K. The temperatures of 302, 328, and 374 K were selected based on experimentally reported thermodynamic parameters of the CheY proteins. Specifically, 302 and 374 K correspond to the temperature of maximum stability (*T*_s_) and the melting temperature (*T*_m_) of the TmY protein, respectively, whereas 328 K corresponds to the melting point of the *B. subtilis* CheY protein (BsY).^16^ The higher temperatures (400 and 450 K) were included to accelerate conformational fluctuations and probe unfolding-related behavior. Because protein unfolding typically occurs on time scales that exceed those accessible to microsecond MD simulations near *T*_m_. Elevated temperatures are commonly employed to enhance sampling and promote unfolding events, as previously demonstrated by Daggett and co-workers.^29^ This strategy allows exploration of thermal destabilization mechanisms within feasible computational times. The distance of *L* = 2.0 nm was employed only for EcY at 450 K, to prevent interactions between periodic images due to the extensive unfolding observed under this condition.
(b) NPT equilibration. Pressure equilibration was performed under the isothermal–isobaric (NPT) ensemble for 100 ps using Parrinello–Rahman’s barostat.^30^ The temperature was kept constant for each case, while the pressure fluctuated around 1 bar, applying a compressibility coefficient of 4.5 × 10^5^ bar^–1^. The resulting densities of the equilibrated systems were approximately 1033.9 ± 4.1 kg·m^–3^ for TmY and 1035.8 ± 4.9 kg·m^–3^ for EcY at 302 K.

The Particle Mesh Ewald (PME) method was employed to compute long-range electrostatic interactions using a Coulomb cutoff of 1.0 nm.^31^ A 1.0 nm cutoff was also applied to evaluate van der Waals interactions. Bond lengths were constrained using the LINCS algorithm,^32^ and an integration time step of 2 fs was applied throughout the simulations.

To ensure reproducibility, each simulation was performed in triplicate at every temperature, resulting in a total of 15 trajectories per protein and 30 independent simulations overall. Each trajectory comprised 5.0 × 10^8^ integration steps with a time step of 2 fs, corresponding to 1 μs of simulation time. System coordinates were recorded every 100 ps, yielding 10,001 saved conformations per trajectory.

### 2.3 Simulation Analysis

Each MD trajectory was analyzed by evaluating several structural and interaction parameters as follows:

(a) Root-mean-square deviation (RMSD). Global backbone fluctuations were quantified using the *rms* module of GROMACS, taking the initial protein conformation (*t* = 0 ns) as the reference structure. RMSD was employed as a standard descriptor to monitor the overall structural deviation of the protein backbone from its initial conformation during the simulations. This metric provides a direct measure of conformational drift and global stability, allowing the identification of temperature-induced structural changes and the assessment of fold preservation over time.
(b) Radius of gyration (*R*_g_). The global compactness of each protein was determined using the *gyrate* module of GROMACS. This parameter provides a quantitative measure of the mass-weighted distribution of atoms around the protein’s center of mass and is commonly used to assess changes in overall compactness during MD simulations. Variations in *R*_g_ were used to identify temperature-dependent expansion or compaction events, offering insight into folding stability and early stage of thermal unfolding
(c) Fraction of native contacts (*Q*). The fraction of native contacts was employed as a global structural metric to monitor overall fold preservation and thermal stability along the simulations. By construction, *Q* captures the collective integrity of the native contact network rather than localized structural rearrangements, making it particularly suitable for assessing global stability under varying temperature conditions. This parameter was estimated using MDTraj,^33^ based on the Best–Hummer–Eaton model.^34^ Each conformation was compared with the initial structure (*t* = 0 ns), defined as the native conformation. The initial fraction has *Q* = 1.0, while subsequent conformations exhibit lower values (*Q* < 1.0).
(d) Secondary structure content (SS). Secondary structure assignments were obtained using the *dssp* module of GROMACS v2024.1, which implements the Define Secondary Structure of Proteins (DSSP) algorithm.^35^ This analysis enabled a residue-level characterization of α-helices, β-strands, and other SS elements along the trajectories. The temporal evolution and relative populations of SS were analyzed using a custom Python v3.9.0 script (https://www.python.org/downloads/release/python-390/), allowing the identification of temperature-dependent destabilization patterns and providing localized insight into structural integrity that complements the global descriptors.
(e) Root-mean-square fluctuation (RMSF). The flexibility of individual amino acid residues was quantified using the *rmsf* module of GROMACS. RMSF provides a residue-resolved measure of atomic mobility around the average structure, allowing the identification of regions with enhanced conformational fluctuations. This descriptor is particularly useful for detecting localized flexibility changes induced by temperature and for relating dynamic behavior to the loss or preservation of secondary-structure elements, thereby complementing the global stability metrics.
(f) Hydrogen bonds (HB). These interactions were performed using the *hbond* module of GROMACS. A hydrogen bond is defined by a donor–acceptor distance *r*_HB_ ≤ 3.5 Å and a donor-hydrogen-acceptor angle θ_HB_ ≤ 30°. This analysis provides insight into the temperature-dependent stabilization of SS elements and local interaction patterns, complementing the assessment of electrostatic interactions and overall structural stability.
(g) Salt bridges (SB). Salt bridges were identified using the GetContacts program (https://getcontacts.github.io/), by monitoring distance-based electrostatic interactions between oppositely charged residues (Lys/Arg and Asp/Glu) throughout the MD trajectories, following the geometric criterion proposed by Barlow and Thornton, in which a salt bridge is defined when the distance between charged atoms is *r*_SB_ ≤ 4.0 Å.^36^ For each replica, salt-bridge frequencies were calculated as the fraction of simulation frames in which the interaction was present. The final frequency reported for each salt bridge corresponds to the average value across the three independent replicas, and only interactions with an average frequency ≥0.3 (*f*_SB_ ≥ 0.30) were considered as formed, even if individual replicas exhibited lower frequency.

Taken together, the combination of global and local structural descriptors employed in this work provides a comprehensive framework to assess the protein stability and thermal responses. Global metrics, such as RMSD, *R*_g_, and *Q*, were used to monitor overall fold preservation and compactness as a function of the temperature, whereas local descriptors, including RMSF, SS analysis, and SB interactions, were used to capture region-specific flexibility and interaction patterns. This multiscale approach enables a robust interpretation of the molecular mechanisms underlying the enhanced thermal stability of TmY relative to that of EcY across the investigated temperature range.

## 3 Results and Discussion

### 3 1 Structural Analyses

As stated earlier, the minimum distance between the outermost atoms of the EcY protein and the edge of the simulation box was set to *L* = 2.0 nm at 450 K, since at *L* = 1.0 nm, the protein interacts with its periodic images. To verify that an increase in box size does not alter the overall trajectory trends, additional simulations were performed at each temperature using a *L* of 2.0 nm. Figures S1 and S2 in the Supporting Information show the time evolution of the RMSD and *R*_g_ parameters at 302, 328, 374, and 400 K for both distances (*L* = 1.0 and 2.0 nm). As shown, these structural behaviors remain consistent regardless of box dimensions. Therefore, it is valid to compare the EcY trajectories at 302, 328, 374, and 400 K obtained with *L* = 1.0 nm to those at 450 K with *L* = 2.0 nm, even though the latter systems are larger and require more computational time to integrate the equations of motion.

The time series of the *C*_α_ root-mean-square deviation, radius of gyration, and fraction of native contacts for both TmY and EcY proteins at 302, 374, and 450 K are shown in Figure 2. At 302 K, both proteins appear to be in their folded state, as none of the three structural parameters shows significant deviations (Figure 2a,d,g). However, the mesophilic protein exhibits slightly higher RMSD and *R*_g_ values and lower *Q* values than its thermophilic counterpart.

**Figure 2:**
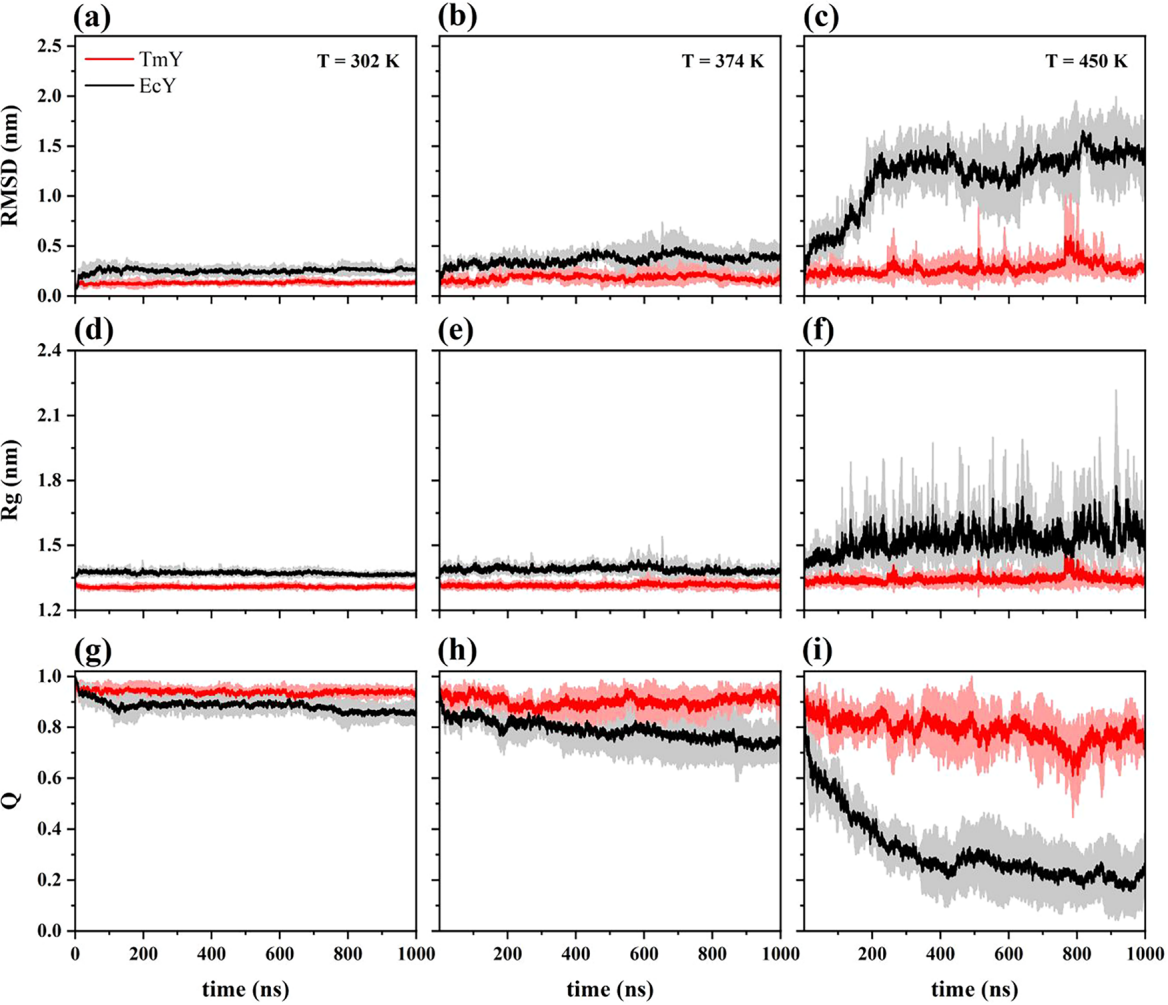
Time series of (a–c) RMSD, (d–f) *R*_g_, and (g–i) *Q* at 302, 374, and 450 K. The solid lines represent the mean values, and the shaded areas indicate the corresponding standard deviations at each time point. Trajectories were obtained from three independent simulations for the TmY (red) and EcY (black) proteins. Complete trajectories are provided in Figures S3, S4, and S5 of the Supporting Information.

At 374 K, both proteins show increases in RMSD and *R*_g_ and a decrease in Q, although these changes are more pronounced in the mesophilic protein (Figure 2b,e,h). The average *R*_g_ value for both proteins remains essentially constant, exhibiting only small fluctuations, as expected. Upon further heating to 450 K, the EcY protein undergoes dramatic structural alterations, as reflected in all three parameters (Figure 2c,f,i). These results indicate that EcY becomes fully unfolded, losing essentially all of its secondary structures. In contrast, the TmY protein undergoes only a partial loss of secondary elements while largely preserving its tertiary structure.

In addition to these structural parameters, the root-mean-square fluctuation behavior and content and temporal evolution of secondary structures were also analyzed. The most relevant observations derived from the RMSD, *R*_g_, *Q*, RMSF, and SS analyses are discussed below.

#### 3.1.1 Root-Mean-Square Deviation

Figure 3 shows the mean and standard deviation values of the RMSD for both proteins at the five analyzed temperatures. In Figure 3a, it is observed that the RMSD values of the EcY protein are higher than those of TmY at all temperatures. For instance, under folding conditions (302 K), TmY exhibits a mean RMSD of 0.131 ± 0.017 nm, whereas EcY shows a mean RMSD of 0.250 ± 0.043 nm. On average, the RMSD of EcY is 0.119 nm greater than that of TmY, meaning that the RMSD of the EcY protein is 90.8% larger. At the first unfolding temperature (400 K), the mean RMSD values of TmY and EcY are 0.184 ± 0.042 and 0.502 ± 0.120 nm, respectively, indicating a mean difference of 0.318 nm; thus, the RMSD of EcY is 172.8% larger than that of TmY. At the second unfolding temperature (450 K), the RMSD of EcY increases substantially to 1.193 ± 0.347 nm, whereas TmY exhibits a slight increase to 0.273 ± 0.093 nm. The mean difference between these values (0.920 nm) indicates that the RMSD of EcY is 337% greater than that of TmY.

**Figure 3:**
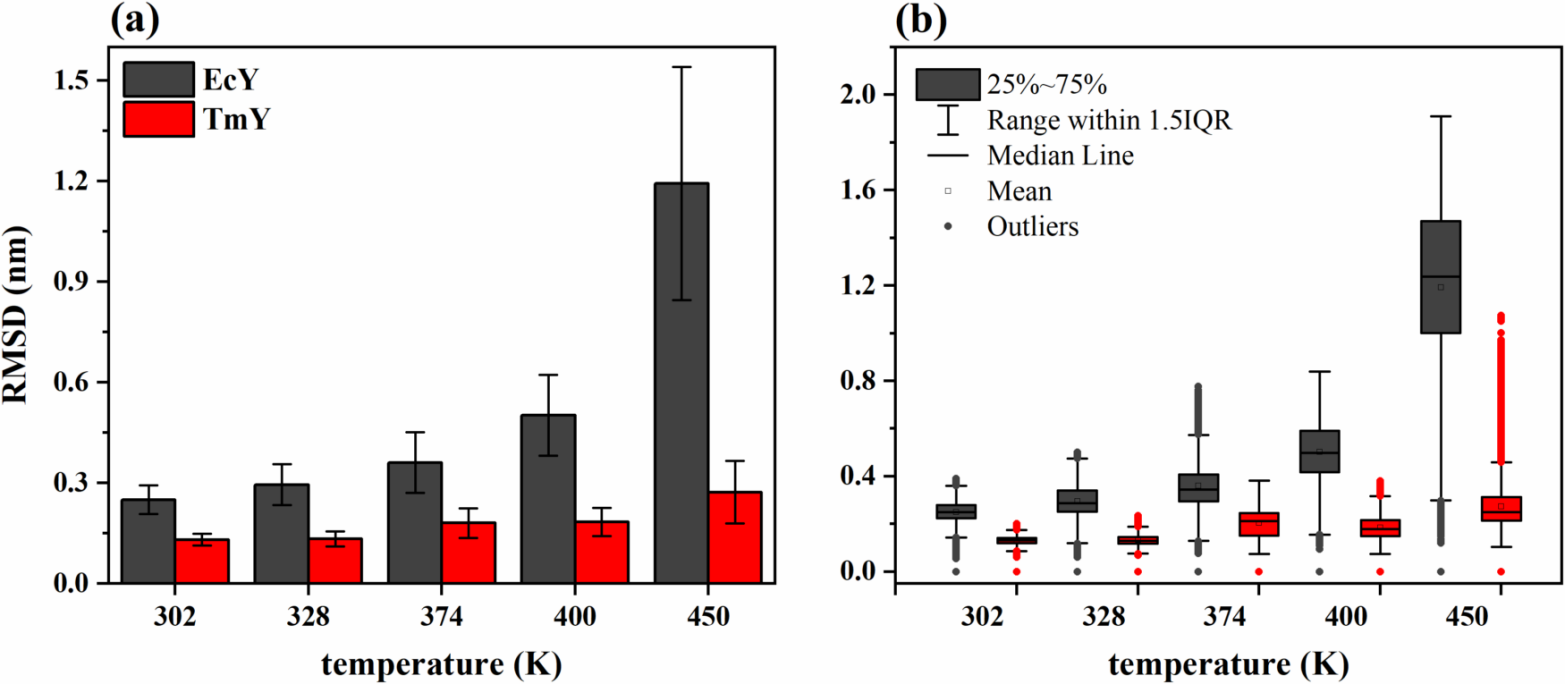
Statistical trends of the RMSD trajectories for TmY and EcY proteins at 302, 328, 374, 400, and 450 K: (a) histograms and (b) boxplots. Table S1 of the Supporting Information shows the corresponding statistical descriptors (mean, standard deviation, and median) calculated from 30,003 values.

Furthermore, from the folded state to the first unfolding temperature, i.e., in the range of 302 to 400 K, the TmY and EcY proteins exhibit increases in RMSD of 0.053 nm (40.5%) and 0.252 nm (100.8%), respectively. This behavior changes drastically for the EcY protein between 302 and 450 K, as the RMSD increases by an average of 0.943 nm (377.2%), whereas the TmY protein shows only a slight change of 0.142 nm (108.4%). These results demonstrate that the mesophilic protein becomes entirely unfolded at 450 K, while the thermophilic protein, although undergoing some structural rearrangements, maintains its stability and resists the destabilizing effects of the temperature.

On the other hand, Figure 3b shows the statistical distributions of the RMSD data. It can be observed that the interquartile range (IQR) of the EcY protein is larger than that of TmY, indicating that the mesophilic protein explores a broader range of structural conformations at each temperature. The IQR increases dramatically for EcY at 450 K, suggesting that the number of sampled conformations also rises. Moreover, the mean value is lower than the median, and the lower whisker is longer than the upper one; therefore, the EcY distribution is left-skewed, meaning that most RMSD values are greater than the mean, or in other words, there are more large RMSD values than small ones. In contrast, the TmY distribution is right-skewed, that is, the mean is higher than the median and the lower whisker is shorter than the upper one. Although TmY exhibits a larger number of outliers, most RMSD values are lower than the mean, or equivalently, there are more small RMSD values than large ones.

#### 3.1.2 Radius of Gyration

The radius of gyration of the TmY protein shows practically no change from 302 to 450 K, increasing on average by only 0.036 nm (2.8%), whereas the EcY protein exhibits a slightly larger variation, with an average increase of 0.151 nm (11.0%) over the same temperature range. Overall, there is little difference in the *R*_g_ behavior between the two proteins. For instance, the average *R*_g_ values of the EcY structures are 0.064 nm (4.9%) and 0.179 nm (13.3%) higher than those of the TmY protein at 302 and 450 K, respectively (Figure 4a).

**Figure 4:**
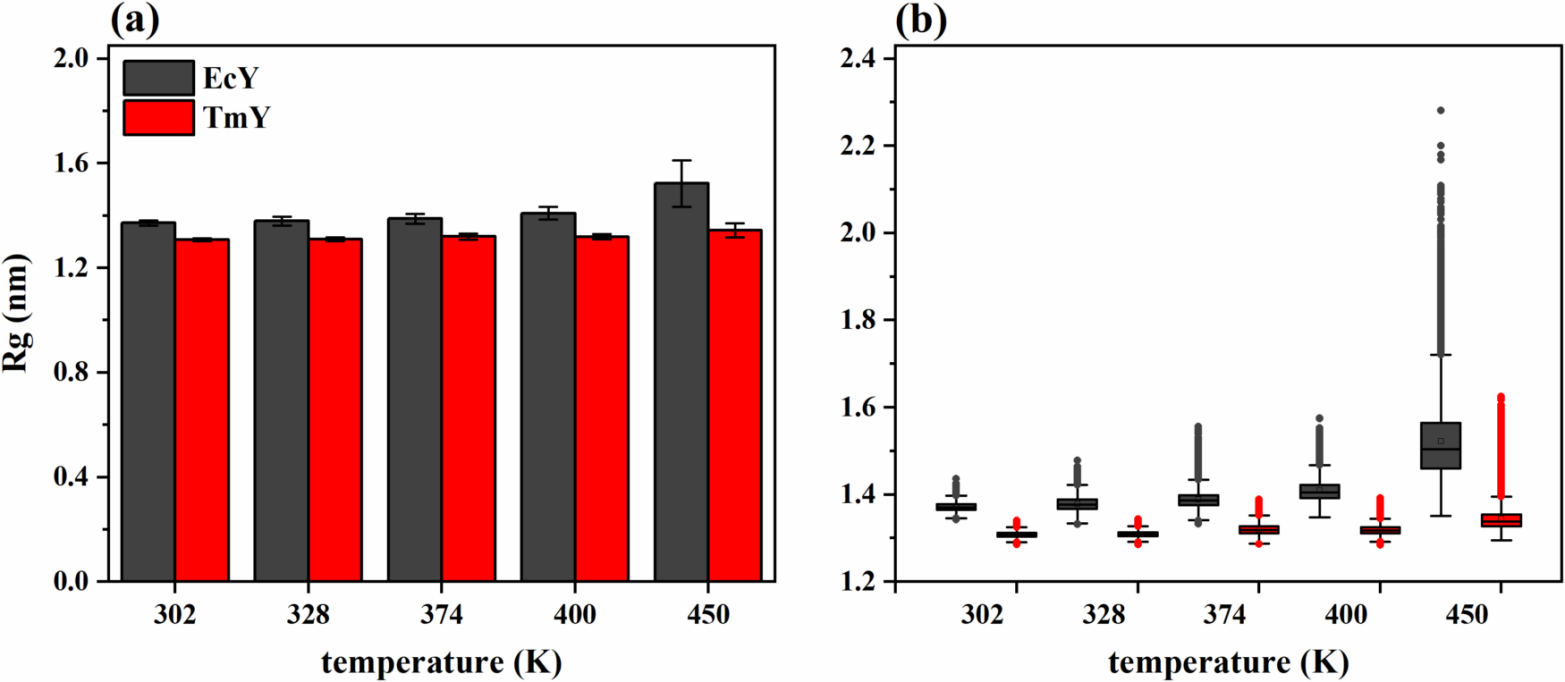
Statistical trends of the *R*_g_ trajectories for TmY and EcY proteins at 302, 328, 374, 400, and 450 K: (a) histograms and (b) boxplots. Table S2 shows the corresponding statistical descriptors (mean, standard deviation, and median) calculated from 30,003 values.

Although no drastic changes are observed in the *R*_g_ values, compared with those seen for RMSD, the boxplots indicate that the mesophilic protein adopts more expanded structures than its hyperthermophilic counterpart as the temperature increases (Figure 4b). The EcY protein exhibits a larger interquartile range (within 1.5 × IQR) and a greater number of outliers than TmY at all five temperatures analyzed. These differences become particularly pronounced at 450 K, indicating that the mesophilic protein explores a broader ensemble of conformational states, thereby triggering a marked increase in the level of structural instability.

The findings obtained from the *R*_g_ calculations are consistent with the trends observed in the RMSD. At elevated temperatures, the unfolding of the EcY protein leads to a greater number of expanded conformations. Hydrophobic interactions among residues tend to maintain a compact core, thereby favoring chain compaction. However, the increase in the kinetic energy promotes structural expansion. In other words, the *R*_g_ measurements reveal the competition between two opposing forces: one that stabilizes the hydrophobic core through hydrophobic interactions and another that drives the structural expansion through kinetic energy.

#### 3.1.3 Fraction of Native Contacts

Figure 5 shows that the mesophilic protein exhibits a lower number of native contacts than the thermophilic one at 302 K, indicating that the native structure of EcY is intrinsically less stable than that of TmY. The fraction of native contacts is 0.939 ± 0.016 for TmY and 0.884 ± 0.031 for EcY. As the temperature increases, *Q* decreases in both proteins; however, the mesophilic protein undergoes a more pronounced loss of native contacts compared to its hyperthermophilic counterpart (Figure 5a). For instance, at 400 K, TmY and EcY show values of 0.898 ± 0.036 and 0.657 ± 0.084; therefore, the proteins lose on average *Q* = 0.041 and *Q* = 0.227, corresponding to reductions of 4.4 and 25.7%, respectively, relative to their folded states. The loss becomes more drastic at 450 K, where TmY retains a *Q* of 0.792 ± 0.073 (a decrease of 0.147, or 15.7%), while EcY drops to 0.311 ± 0.149 (a decrease of 0.573, or 64.8%), reflecting a pronounced reduction of 64.8% in native contacts over the 302–450 K range. Moreover, the difference in the fraction of native contacts between the two proteins increases with temperature. For instance, the average *Q* difference between TmY and EcY at 302 K is 0.055, corresponding to 5.9%. At the unfolding temperatures, the *Q* differences rise to 0.241 (26.8%) at 400 K and 0.481 (60.7%) at 450 K.

**Figure 5:**
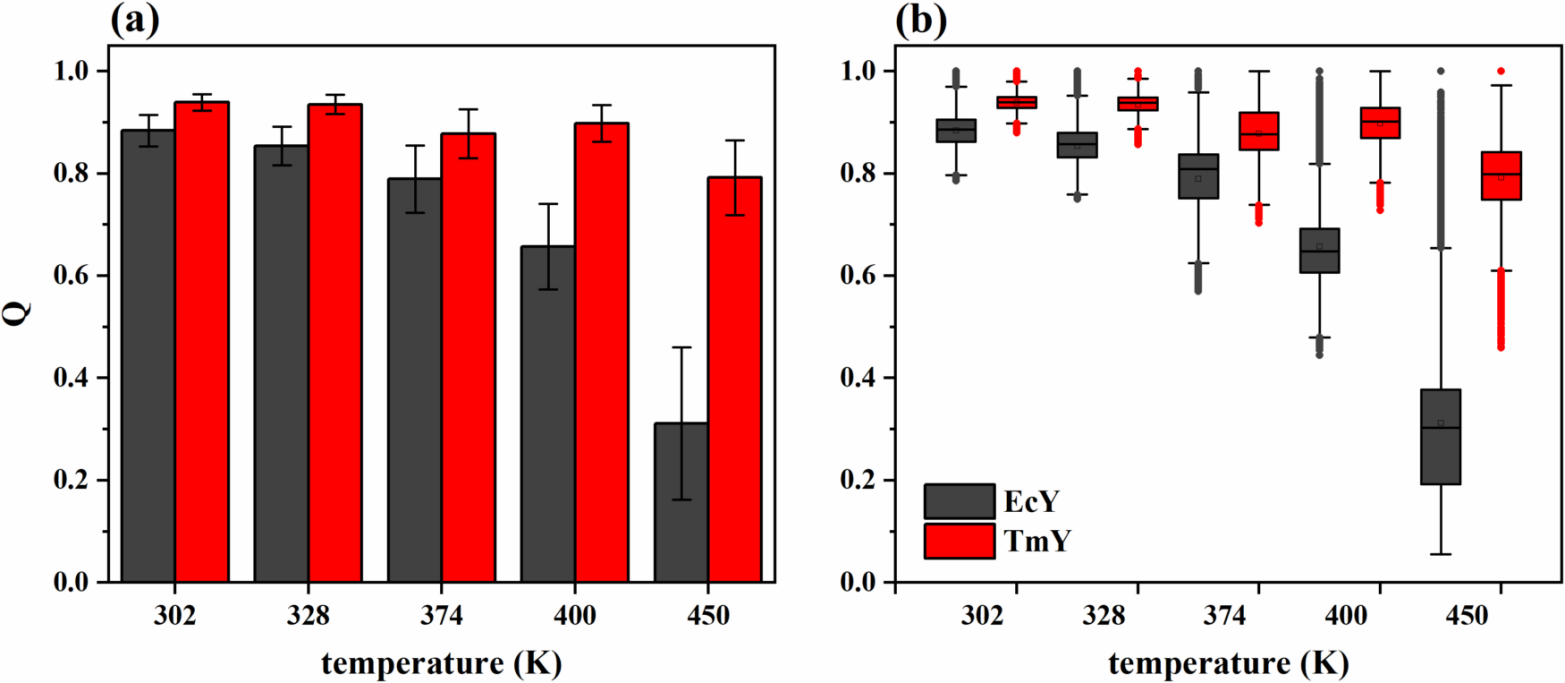
Statistical trends of the *Q* trajectories for TmY and EcY proteins at 302, 328, 374, 400, and 450 K: (a) histograms and (b) boxplots. Table S3 shows the corresponding statistical descriptors (mean, standard deviation, and median) calculated from 30,003 values.

The boxplots (Figure 5b) show that the distributions of both proteins are symmetric at low temperatures (302 and 328 K). At higher temperatures (400 and 450 K), the mesophilic protein exhibits right-skewed distributions, whereas the hyperthermophilic protein shows a slight left skew. These trends indicate that EcY tends toward lower values of native contacts, while TmY tends to maintain higher ones. Another noteworthy observation is that both proteins display increases in the interquartile range, in the range within 1.5 × IQR, and in the number of outliers; however, these increases are more pronounced for the mesophilic protein. Additionally, EcY exhibits conformations that can lose up to 95% of their native contacts at 450 K, as indicated by the lower whisker, reaching *Q* = 0.05. This result suggests an almost complete loss of native contacts at this temperature, consistent with RMSD and *R*_g_ analyses that indicate the full unfolding of the structure.

The behavior observed for the fraction of native contacts is fully consistent with the trends identified in the RMSD and *R*_g_ analyses. In the case of TmY, the preservation of a high *Q* explains the absence of large global structural fluctuations and the maintenance of a compact conformation, even at elevated temperatures, preventing extensive unfolding. In contrast, EcY exhibits a pronounced loss of native contacts as temperature increases, which correlates with the larger global fluctuations and marked structural expansions reflected in the RMSD and *R*_g_ profiles, ultimately leading to complete unfolding at 450 K. This interpretation is further supported by the structural evolution shown in Figure S6 of the Supporting Information, where EcY progressively loses secondary structure elements with increasing temperature, reaching a fully unfolded state at 450 K, whereas TmY largely preserves its native fold.

To gain deeper insight into how these structural changes affect the organization of the TmY and EcY proteins, the evolution of the secondary structure elements was subsequently analyzed as follows.

#### 3.1.4 Secondary Structures

As shown in Figure 1c, the TmY protein consists of 55, 25, 3, and 35 residues corresponding to the α-helix, β-strand, 3_10_-helix, and random coil structures, representing 46.61, 21.19, 2.54, and 29.66% of the total residues, respectively. Similarly, the EcY protein comprises 58 (45.31%), 22 (17.19%), and 48 (37.50%) residues forming α-helix, β-strand, and random coil structures, respectively. It is worth noting that random coil structures refer to those residues that do not adopt α-helix or β-strand conformations.

In this work, the residues of both proteins were classified into three structural categories to simplify the secondary structure assignments generated by the GROMACS program: (1) α-helix, which includes α-helix, 3_10_-helix, and π-helix conformations; (2) β-strand, which comprises only the extended strand arrangement; and (3) random coil, which encompasses all remaining structures, namely, bends, turns, loops, and β-bridges. Considering these definitions, at 302 K (i.e., under folded conditions), the simulations reveal that the TmY protein contains 40.13 ± 3.10% of α-helices and 20.85 ± 1.31% of β-strands, whereas the EcY protein exhibits 32.17 ± 3.22% of α-helices and 16.98 ± 0.95% of β-strands. These results indicate that, during the simulations, the α-helical content decreases on average by 9.02% in TmY and 13.14% in EcY compared with their X-ray structures. In contrast, the β-strand content remains nearly unchanged, showing average values comparable to those observed in the experimental structures.

This behavior is likely due to the presence of ILV clusters formed by 10 residues from the β-strands in both proteins, corresponding to 40.0 and 45.5% of the residues within these secondary structures in TmY and EcY, respectively. In contrast, 12 (21.8%) residues in TmY and 14 (24.1%) residues in EcY form ILV clusters within α-helical regions. It is worth noting that the side chains of isoleucine (I), leucine (L), and valine (V) residues contribute significantly to the hydrophobic effect in globular proteins.^37^ Therefore, the ILV clusters provide greater structural stability to β-strands than to α-helices in both proteins. Figure S7 of the Supporting Information displays a representative snapshot of the ILV clusters identified in the TmY and EcY proteins.

##### 3.1.4.1 α-Helix Content

Figure 6 summarizes the temperature dependence of the α-helical content for both proteins. Across the entire temperature range, the thermophilic protein TmY consistently preserves a higher fraction of the α-helical structure than its mesophilic counterpart EcY, indicating a greater resistance of its secondary-structure elements to thermal perturbation. As temperature increases, the two proteins experience progressive loss of α-helices; however, this loss is markedly more pronounced in EcY, reflecting a reduced ability to maintain local structural order under destabilizing conditions (Figure 6a).

**Figure 6:**
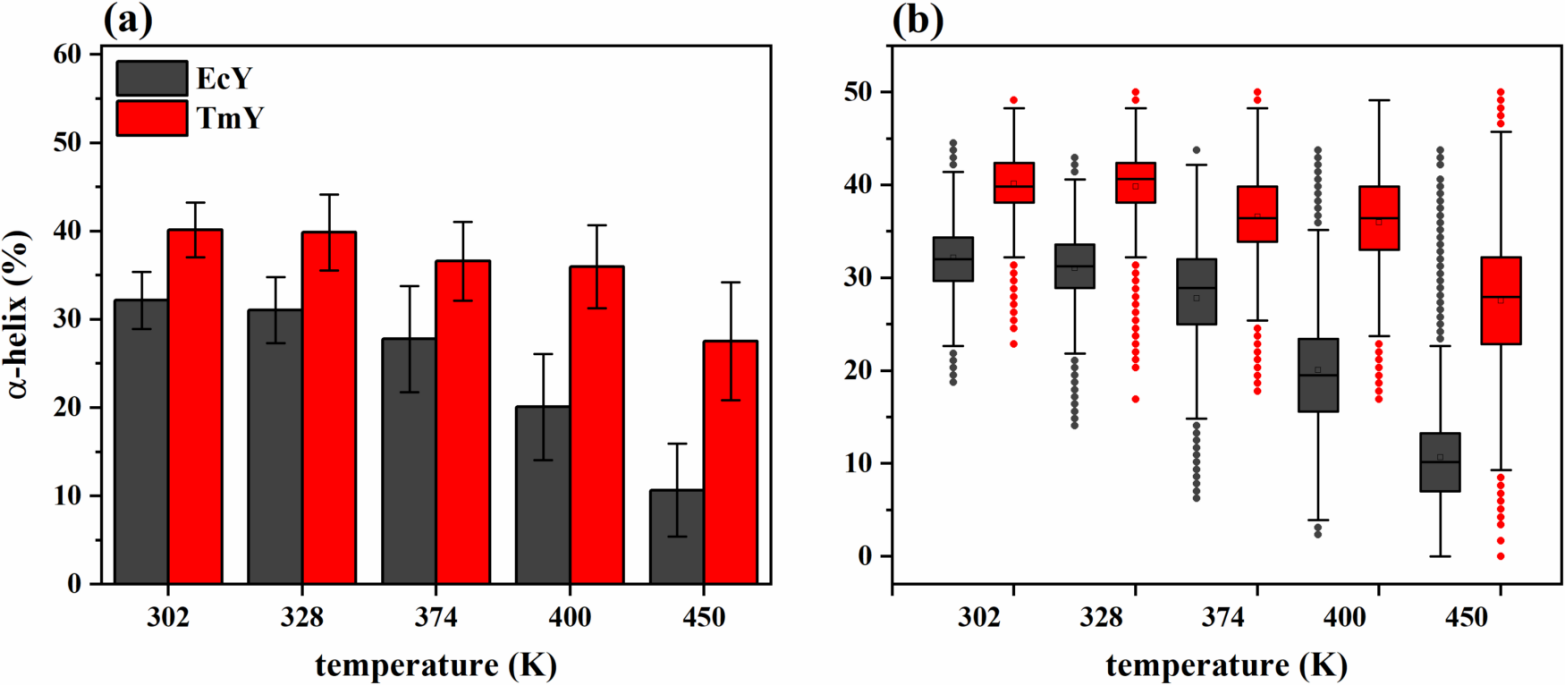
Statistical trends of the α-helix percentages for TmY and EcY proteins at 302, 328, 374, 400, and 450 K: (a) histograms and (b) boxplots. Table S4 of the Supporting Information shows the corresponding statistical descriptors (mean, standard deviation, and median) calculated from 30,003 values.

From a mechanistic perspective, the enhanced persistence of α-helical elements in TmY contributes to the preservation of a compact fold as helices act as rigid scaffolds that limit large-amplitude backbone fluctuations. In contrast, the more extensive helix destabilization observed in EcY promotes local unfolding events that propagate into global structural expansion, consistent with the larger RMSD and *R*_g_ values as well as the concomitant decrease in *Q* observed at elevated temperatures.

Analysis at the level of individual helices supports this interpretation. In EcY, the terminal helices, particularly those located near the N- and C-termini (α_1_- and α_5_-helices), are more susceptible to thermal disruption, leading to increased flexibility and loss of tertiary contacts. In TmY, these same regions display greater resilience, suggesting that helix stabilization is reinforced by cooperative interactions with the surrounding structural elements, including persistent hydrogen-bond networks and electrostatic interactions. Although certain helices in TmY also weaken at higher temperatures (α_3_- and α_4_-helices), their destabilization does not propagate into complete loss of fold integrity, underscoring the role of distributed stabilization mechanisms rather than reliance on a single secondary-structure element.

Figure 6b shows the box plot distributions of α-helix content for both proteins. At all temperatures, TmY consistently exhibits higher and whiskers median values than EcY, reflecting a more robust preservation of α-helical structure. EcY displays broader interquartile ranges (1.5 × IQR) between 302 and 400 K, indicating increased structural heterogeneity and a higher susceptibility to local helix destabilization. At 450 K, this behavior becomes more pronounced: EcY protein exhibits a right-skewed distribution dominated by low α-helix content, with only few transient conformations retaining higher helical fractions, as outliers appear at high percentages. Conversely, TmY maintains a broader distribution of α-helix preservation even at this extreme temperature, with occasional low-percentage outliers but a substantial fraction of conformations remaining partially structured. These statistical trends reinforce the notion that the mesophilic protein undergoes a more extensive and heterogeneous loss of α-helical elements upon thermal stress, whereas the thermophilic homologue retains a greater degree of secondary-structure integrity, consistent with its enhanced thermal resilience. Detailed helix-specific percentages and temperature-dependent trends of both proteins are provided in Table S5 and Figure S8 of the Supporting Information.

##### 3.1.4.2 β-Strand Content

The thermal response of β-strands differs markedly from that observed for α-helices, particularly in the thermophilic protein. In TmY, the β-strand content remains largely preserved across the entire temperature range, in contrast to the gradual loss of the α-helical structure, indicating that the β-sheet core constitutes a particularly robust structural element under thermal stress. Conversely, in the mesophilic EcY protein, β-strands display temperature-dependent destabilization that parallels the behavior of its α-helices, reflecting a more generalized loss of secondary-structure integrity upon heating.

This distinction is clearly captured in the statistical distributions shown in Figure 7. While EcY exhibits a progressive loss of β-strand content accompanied by a broadening of the corresponding distributions at elevated temperatures (most pronounced at 450 K), TmY maintains narrow and relatively stable distributions, even under extreme thermal conditions (Figure 7a,b). The increased dispersion observed for EcY indicates enhanced structural heterogeneity and partial disruption of the β-sheet architecture, whereas the persistence of compact distributions in TmY reflects sustained β-sheet integrity.

**Figure 7:**
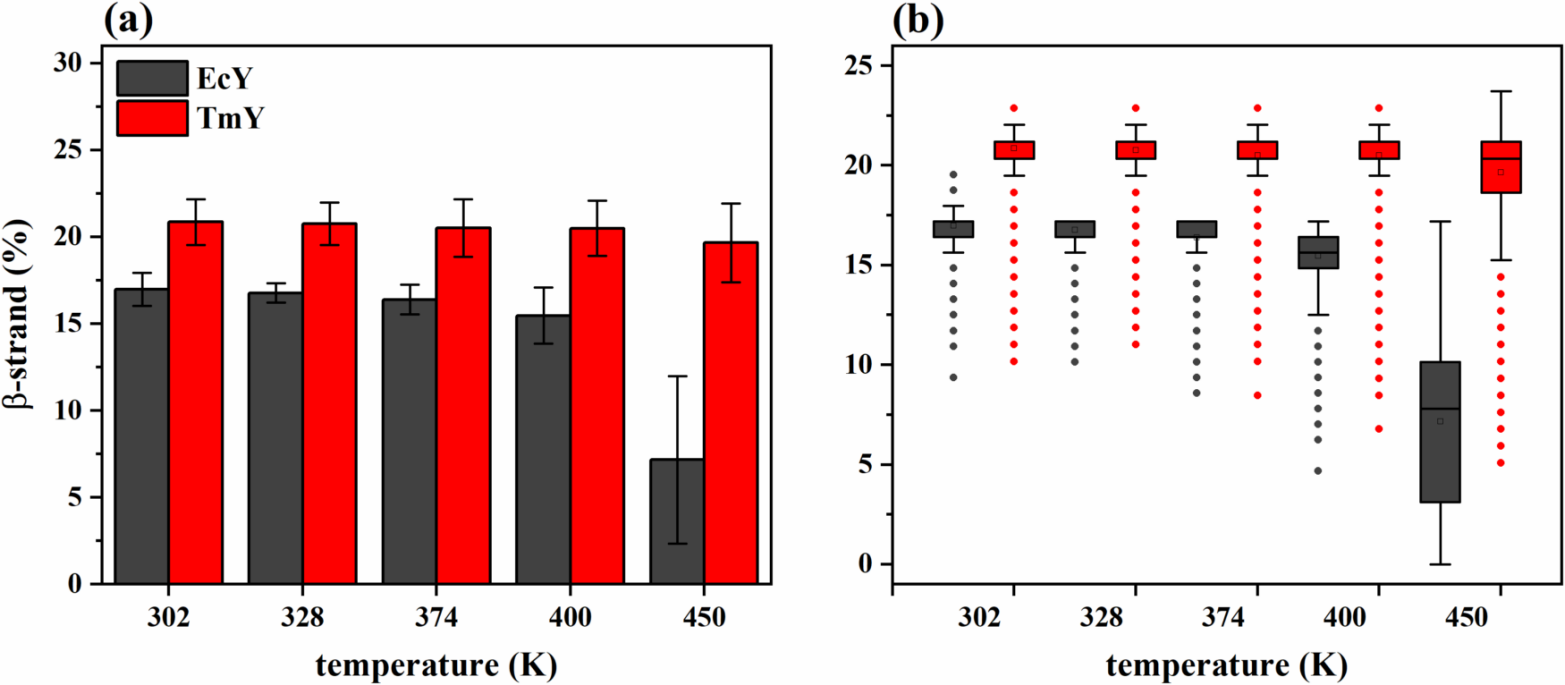
Statistical trends of the β-strand percentages for TmY and EcY proteins at 302, 328, 374, 400, and 450 K: (a) histograms and (b) boxplots. Table S6 shows the corresponding statistical descriptors (mean, standard deviation, and median) calculated from 30,003 values.

Residue-level analysis further highlights these contrasting behaviors. In TmY, all five β-strands remain highly stable, including those located within the hydrophobic core (β_1_, β_3_, and β_4_) as well as the more solvent-exposed strands (β_2_ and β_5_),^10^ suggesting effective protection of the β-sheet scaffold. In contrast, EcY exhibits a progressive weakening of multiple five β-strands, including those forming the hydrophobic core, which become increasingly destabilized as the temperature rises. This loss of β-sheet stability likely contributes to the exposure of the protein core, facilitating global unfolding and reinforcing the trends observed in RMSD, *R*_g_, and native-contact analyses. Detailed strand-specific percentages and temperature-dependent trends of both proteins are provided in Table S7 and Figure S9 of the Supporting Information.

Taken together, these results indicate that preservation of the β-sheet architecture, together with a larger α-helix stability, plays a central role in the superior thermal resilience of TmY. In contrast, the mesophilic EcY protein undergoes a concomitant destabilization of both α-helices and β-strands, leading to a progressive collapse of the secondary-structure framework that accelerates thermal unfolding. These trends are fully consistent with the secondary-structure profiles shown in Figures S10, S11, and S12 of the Supporting Information, which demonstrate that EcY loses a substantially larger fraction of ordered secondary structures than TmY, particularly at 400 and 450 K. At 450 K, EcY exhibits near-complete disruption of its C-terminal domain, with only residual elements of β_1_-, β_2_-, and β_3_-strands and α_2_- and α_3_-helices persisting in the N-terminal domain, mainly in simulations 1 and 3. By contrast, most secondary-structure elements in TmY remain intact across the entire temperature range, with only minor perturbations in the α_3_- and α_4_-helices. Notably, the β_1_–β_4_ strands and the α_1_, α_2_-, and α_5_-helices are largely preserved even at 450 K, indicating that although the C-terminal domain is also affected by temperature, its destabilization is considerably less pronounced than in EcY.

#### 3.1.5 Root-Mean-Square Fluctuation

While the global structural descriptors (RMSD, *R*_g_, and *Q*) provide clear evidence of differential thermal stability between TmY and EcY, they do not reveal which specific regions of the proteins are responsible for these trends. To gain residue-level insight into the localized flexibility underlying the observed global behavior, we next examined the root-mean-square fluctuation profiles of both proteins across the studied temperature range.

Figure 8 shows the residue-wise root-mean-square fluctuation profiles of TmY and EcY at the five analyzed temperatures. As shown in Figure 8a, residues of TmY exhibit low average RMSF values, with maxima of approximately 0.25 nm at 302 and 328 K. This behavior indicates limited flexibility across both ordered secondary-structure elements and disordered regions, consistent with the preservation of structural stability under these thermal conditions.

**Figure 8:**
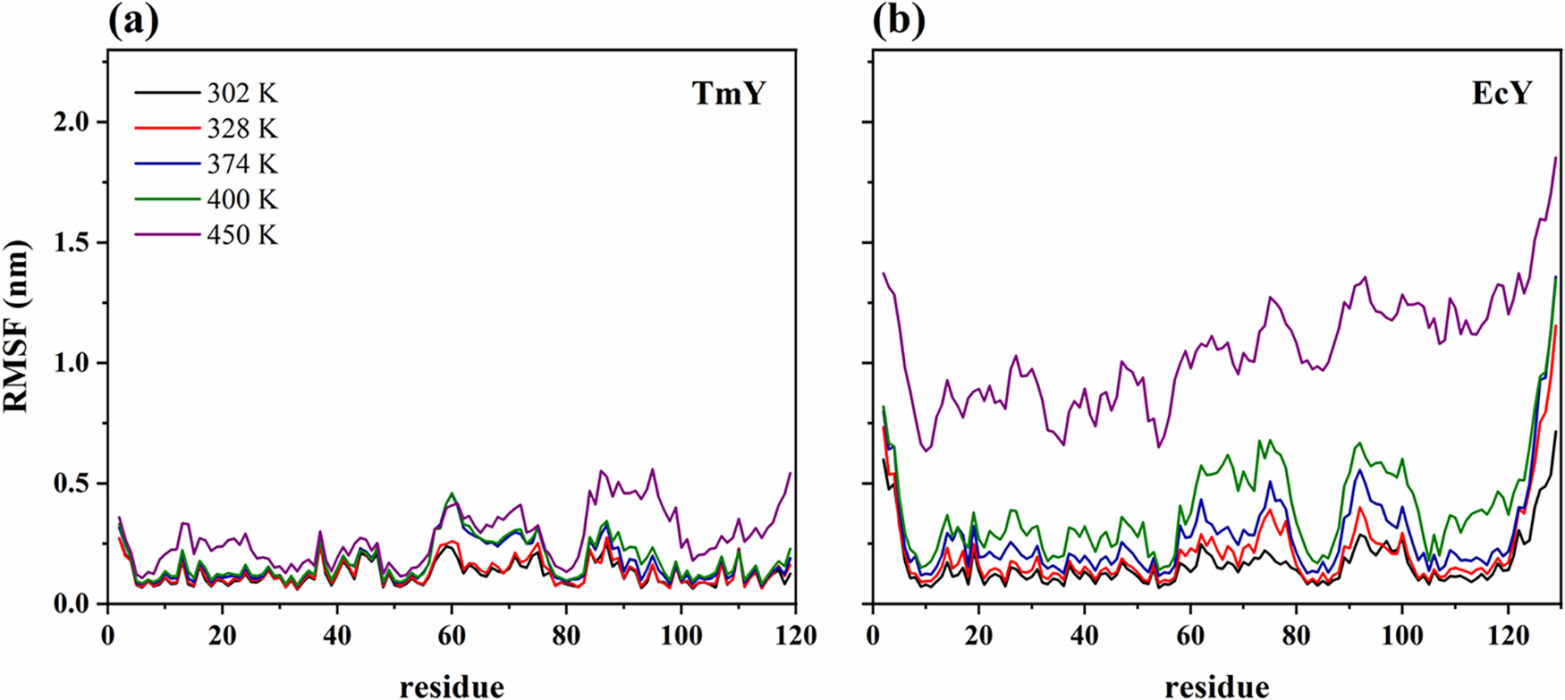
RMSF profiles of (a) TmY and (b) EcY proteins at the five analyzed temperatures. Each point represents the average RMSF value over the three replicas. The RMSF profiles of the individual replicas are provided in Figure S13 of the Supporting Information.

At higher temperatures, the increased flexibility becomes localized to specific regions. In particular, residues 57–75 display enhanced fluctuations, reaching values between 0.30 and 0.40 nm at 374 and 400 K. This segment includes the γ-turn loop (residues 55–61), a highly conserved motif among CheY proteins that contributes a ligand to the metal-binding site and lies adjacent to the active-site residue Asp54.^9–11^ The same interval also contains the α_3_-helix (residues 62–72), which begins to lose structural stability at temperatures ≥374 K (see Figure S8c of the Supporting Information).

At 450 K, residues 83–99 and the two C-terminal residues exhibit RMSF values in the range of 0.40–0.55 nm, consistent with the loss of structural integrity of the α_4_-helix (residues 87–95). Conversely, residues in the N-terminal domain remain comparatively rigid across all temperatures, indicating that both secondary-structure elements and connecting loops preserve their stability, even at 450 K. For instance, the reduced flexibility of the initial loop can be associated with the presence of a stabilizing salt bridge between Arg4 and Glu28.

In contrast, Figure 8b shows that EcY exhibits a markedly higher flexibility than TmY, with multiple regions displaying enhanced RMSF values throughout the structure. Notably, increased fluctuations are observed in residues 57–79, which encompass the γ-turn loop and the α_3_-helix (residues 65–74), residues 88–103 corresponding to the α_4_-helix (residues 92–100), and residues 109–129 containing the α_5_-helix (residues 113–127). These observations are consistent with the structural analyses discussed above, which indicate that these three helices progressively lose their secondary-structure identities as the temperature increases.

Additionally, residues 2–4 in the N-terminal loop display RMSF values exceeding 0.50 nm, reflecting pronounced local flexibility, while a sharp increase in fluctuations is observed toward the C-terminal region. At 450 K, EcY exhibits uniformly high RMSF values, ranging from approximately 0.6 to 1.9 nm, indicating extensive flexibility across the entire protein and consistent with a fully unfolded state under these extreme thermal conditions.

These observations indicate that in both proteins the N-terminal domain exhibits higher rigidity, whereas the C-terminal domain is comparatively more flexible. The enhanced rigidity of the N-terminal region can be largely attributed to the presence of stabilizing electrostatic interactions, particularly salt bridges and salt-bridge networks, which contribute to restraining local fluctuations. The role of these interactions in maintaining structural integrity under increasing temperature is discussed in detail in the corresponding subsection.

### 3.1 Molecular Interactions

#### 3.2.1 Hydrogen Bonds

In this study, two types of hydrogen bond interactions were analyzed: (a) those formed between atoms of residues buried within the protein interior, denoted as HBpp, and (b) those formed between protein residues and water molecules, denoted as HBps. Figures 9 and 10 show the statistical trends of HBpp and HBps, respectively, across the range of temperatures studied. As shown, both interaction types decrease in number for the two proteins as the temperature increases. This behavior arises from the increase in kinetic energy of the system, which promotes the disruption of these weak noncovalent bonds, as further discussed below. Moreover, note that these interactions may undergo rearrangements due to structural motions induced by temperature changes. In other words, any HBpp or HBps interaction is considered in the calculations, even if it is not part of the original network, since hydrogen bonds between residue atoms or between residue and solvent atoms can break and reform throughout the simulations.

**Figure 9:**
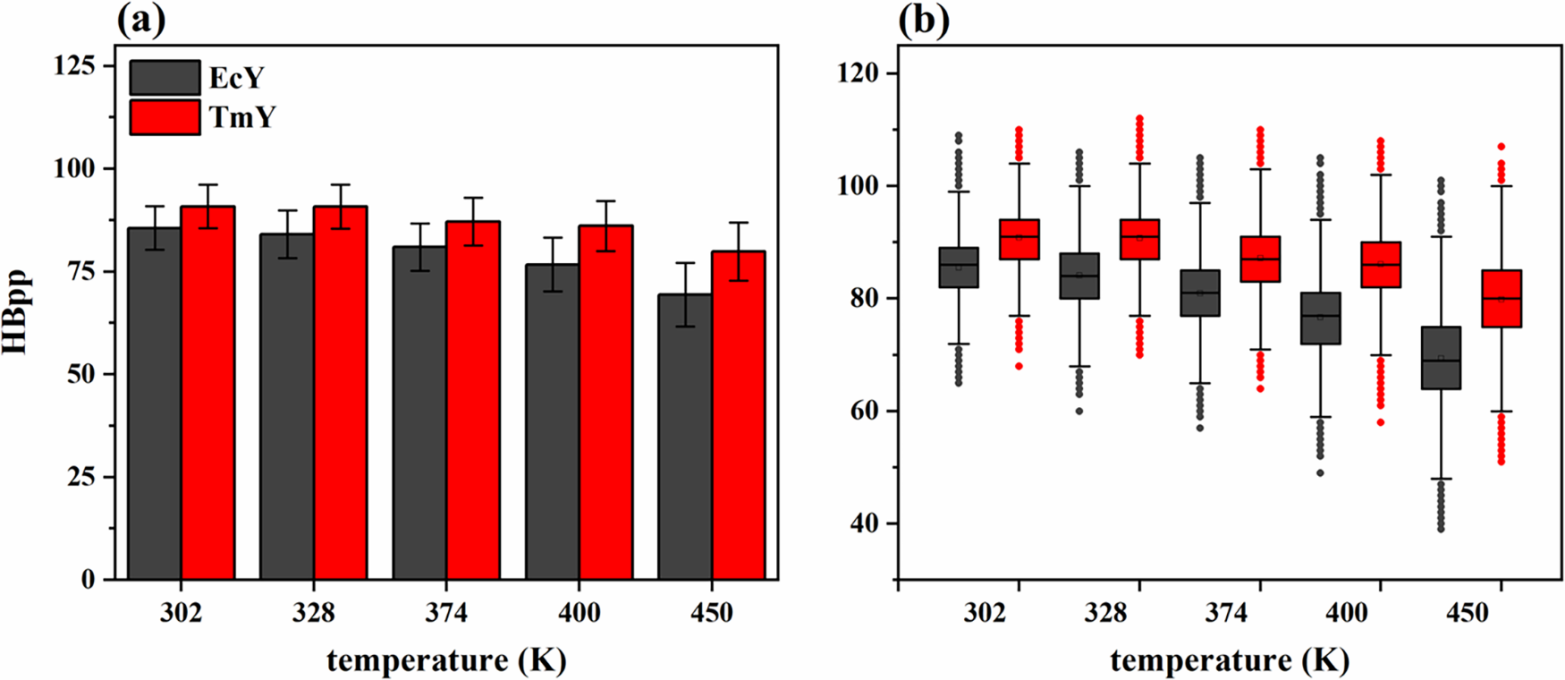
Statistical trends of the number of HBpp for TmY and EcY proteins at 302, 328, 374, 400, and 450 K: (a) histograms and (b) boxplots. Table S8 of the Supporting Information shows the corresponding statistical descriptors (mean, standard deviation, and median) calculated from 30,003 values.

**Figure 10:**
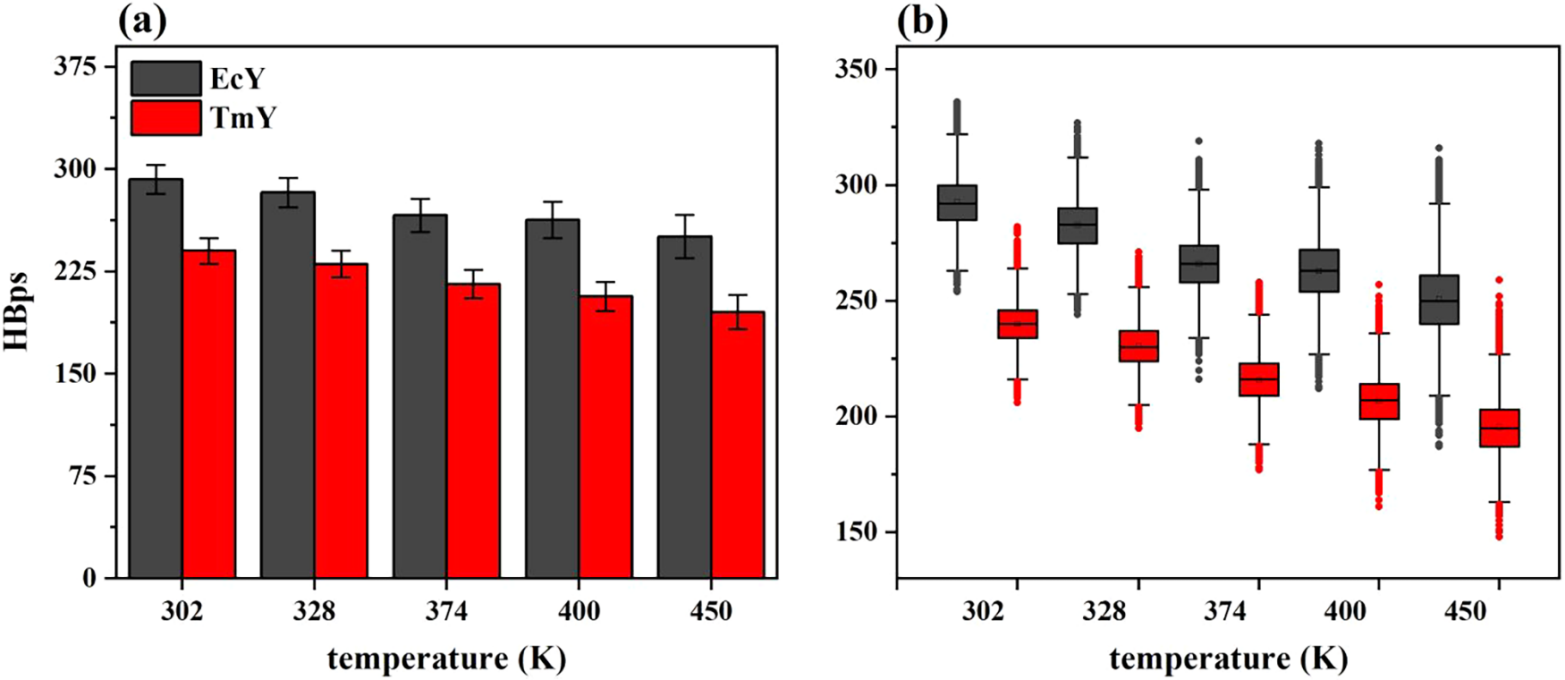
Statistical trends of the number of HBps for TmY and EcY proteins at 302, 328, 374, 400, and 450 K: (a) histograms and (b) boxplots. Table S9 of the Supporting Information shows the corresponding statistical descriptors (mean, standard deviation, and median) calculated from 30,003 values.

Both proteins contain a similar number of polar residues (either charged or uncharged), with 49 residues in TmY and 54 in EcY, corresponding to 41.5 and 42.2% of their total residues, respectively. Thus, based on the comparable chemical composition of polar residues, the two proteins are expected to form a similar number of HBpp interactions in the folded state. However, at 302 K, TmY protein forms slightly more HBpp than its mesophilic counterpart, with mean values of 90.8 ± 5.3 and 85.6 ± 5.3, respectively, reflecting a small difference of approximately 5.2 hydrogen bonds. This trend persists across all temperatures, as illustrated by the histograms and boxplots in Figure 9. Notably, the mesophilic protein exhibits a greater loss of HBpp interactions upon thermal unfolding: at 450 K, TmY and EcY retain mean values of 79.9 ± 7.1 and 69.4 ± 7.8, respectively. This corresponds to a decrease of 10.9 (13.7%) and 16.2 (23.3%) HBpp between 302 and 450 K. These results indicate that the HBpp network in TmY is more resilient to thermal perturbation than that of EcY, as shown in Figure 9a and summarized in Table S8 of the Supporting Information.

Figure 10a shows that the EcY protein exhibits a greater number of HBps than the TmY protein across all five temperatures studied. This observation suggests that the mesophilic protein has a larger proportion of polar residues exposed to the solvent compared with its hyperthermophilic counterpart. At 302 K, EcY forms a mean of 292.6 ± 10.5 HBps, whereas TmY forms 240.1 ± 9.6 HBps, indicating that EcY establishes approximately 21.8% more protein–solvent hydrogen bonds. In addition, both proteins lose a comparable number of these interactions as the temperature increases from 302 to 450 K: TmY and EcY lose 44.8 and 41.9 HBps, corresponding to decreases of 23.0 and 16.7%, respectively. Therefore, although the hyperthermophilic protein loses a slightly higher relative percentage of HBps (6.3%), the mesophilic protein maintains a consistently higher absolute number of solvent-exposed hydrogen bonds throughout the temperature range.

Figures 9b and 10b show that both proteins exhibit approximately symmetric distributions over the temperature range from 302 to 450 K. The interquartile ranges 1.5 × IQR increase as temperature rises; however, the mesophilic protein exhibits interquartile ranges that are equal to or slightly larger than those of the hyperthermophilic protein, highlighting greater variability and dispersion in the number of HBpp and HBps interactions in EcY. This behavior can be directly related to the trend observed for the global structural descriptors, particularly with the radius of gyration. As the EcY protein progressively loses compactness at elevated temperatures, increased solvent exposure accompanies the partial unfolding, leading to weakening and higher instability of both internal hydrogen bonds and protein–solvent hydrogen bonds. In contrast, the relatively stable *R*_g_ values observed for TmY indicate preservation of a compact fold, which limits solvent penetration and contributes to the maintenance of a more stable hydrogen-bonding network. These results suggest that differences in hydrogen-bond dynamics between the two proteins are mechanistically linked to their distinct degrees of structural compactness and thermal resilience.

The hydrogen-bond interactions, particularly those buried within the protein core (HBpp), progressively weaken or are lost as the temperature increases, a behavior that is especially pronounced in the EcY protein. Conversely, these interactions remain comparatively more persistent in TmY, suggesting the presence of additional stabilizing mechanisms. This enhanced resistance may arise, in part, from a cooperative effect among hydrogen bonds, given the slightly higher number of HBpp interactions in TmY, as well as from the synergistic contribution of other noncovalent forces. In this context, hydrogen bonds alone are insufficient to fully account for the remarkable thermal stability of the hyperthermophilic protein. Rather, their persistence likely depends on cooperative stabilization with stronger electrostatic interactions, particularly salt bridges, whose role in maintaining structural integrity at elevated temperatures is analyzed in the following section.

#### 3.2.2 Salt Bridges

In line with the cooperative stabilization mechanisms discussed above, we next examined the role of electrostatic interactions by analyzing salt bridges using the GetContacts program, adopting an average frequency threshold of 0.3 as the formation criterion, as described in the Materials and Methods section. This threshold indicates that on average an interaction between pairs of charged residues is present for at least 30% of the simulation time. Additionally, the following criteria were applied to classify the degree of stability of this interaction.

As shown in Table 1, if the average frequency of a salt bridge (*f*_SB_) is equal to or greater than 0.75, it is considered highly stable, i.e., the charged residue pairs interact with each other, on average, for at least 75% of the simulation time, maintaining the cutoff distance of *r*_SB_ ≤ 4 Å. Conversely, a salt bridge with a frequency lower than 0.30 is regarded as negligible or not formed since its occurrence is below 30% throughout the trajectory. Meanwhile, salt bridges exhibiting moderate and low stability follow these same criteria, according to the intervals specified in the table.

**Table 1:** Classification of the Stability of Salt Bridges

**frequency** (***f***_**SB**_)	**stability**
*f*_SB_ ≥ 0.75	high
0.50 ≤ *f*_SB_ < 0.75	moderate
0.30 ≤ *f*_SB_ < 0.50	low
*f*_SB_ < 0.30	negligible

Building on the stability criteria outlined above, Tables 2 and 3 summarize the intra- and intermolecular salt bridges identified in this research. Each value represents the average over three independent simulations at the five temperatures examined. For the sake of clarity, the secondary structures connected by each salt bridge are also indicated.

**Table 2:** Average Formation Frequencies of the Intra- and Intermolecular Salt Bridges in EcY Protein

		**temperature (K)**				
**residue pairs**	**secondary structures**	**302**	**328**	**374**	**400**	**450**
Arg22–Glu35	α_1_–β_2_	0.980	0.956	0.859	0.768	0.272
Asp41–Lys45	α_2_	0.964	0.951	0.912	0.898	0.695
Asp57–Lys109	β_3_–β_5_/α_5_	0.812	0.727	0.515	0.434	0.173
Asp12–Lys109	β_1_/α_1_–β_5_/α_5_	0.636	0.527	0.215	0.092	0.023
Arg18–Glu35	α_1_–β_2_	0.634	0.718	0.783	0.804	0.197
Arg18–Glu37	α_1_–β_2_/α_2_	0.398	0.346	0.418	0.451	0.167
Glu89–Lys91	β_4_/α_4_	0.375	0.334	0.219	0.179	0.165
Glu67–Lys70	α_3_	0.300	0.348	0.412	0.267	0.372

**Table 3:** Average Formation Frequencies of the Intra- and Intermolecular Salt Bridges in the TmY Protein

		**temperature (K)**				
**residue pairs**	**secondary structures**	**302**	**328**	**374**	**400**	**450**
Asp9–Lys104	β_1_/α_1_–β_5_/α_5_	0.981	0.987	0.979	0.970	0.486
Asp54–Lys104	β_3_–β_5_/α_5_	0.948	0.978	0.950	0.944	0.885
Asp20–Lys24	α_1_	0.909	0.881	0.856	0.849	0.740
Arg15–Glu32	α_1_–β_2_	0.682	0.701	0.715	0.712	0.676
Lys19–Glu32	α_1_–β_2_	0.668	0.649	0.618	0.636	0.622
Arg110–Glu113	α_5_	0.662	0.487	0.505	0.488	0.466
Asp49–Lys77	α_2_/β_3_–α_3_/β_4_	0.560	0.533	0.453	0.482	0.475
Asp100–Lys117	β_5_–α_5_	0.554	0.593	0.625	0.630	0.524
Asp100–Arg110	β_5_–α_5_	0.491	0.605	0.714	0.741	0.570
Arg37–Glu68	α_2_–α_3_	0.468	0.501	0.372	0.383	0.173
Glu92–Lys95	α_4_	0.427	0.462	0.523	0.552	0.346
Arg4–Glu28	β_1_–β_2_	0.420	0.424	0.422	0.426	0.435
Arg37–Asp64	α_2_–α_3_	0.404	0.517	0.334	0.384	0.318
Glu68–Lys71	α_3_	0.382	0.465	0.469	0.470	0.516
Glu41–Lys44	α_2_	0.362	0.371	0.424	0.432	0.463

A total of eight salt bridges were identified in the folded state of the EcY protein: five intermolecular and three intramolecular interactions (Table 2). Additionally, two salt-bridged networks are present, consisting of one triad and one tetrad. The spatial distributions of these interaction patterns are illustrated in Figure 11.

**Figure 11:**
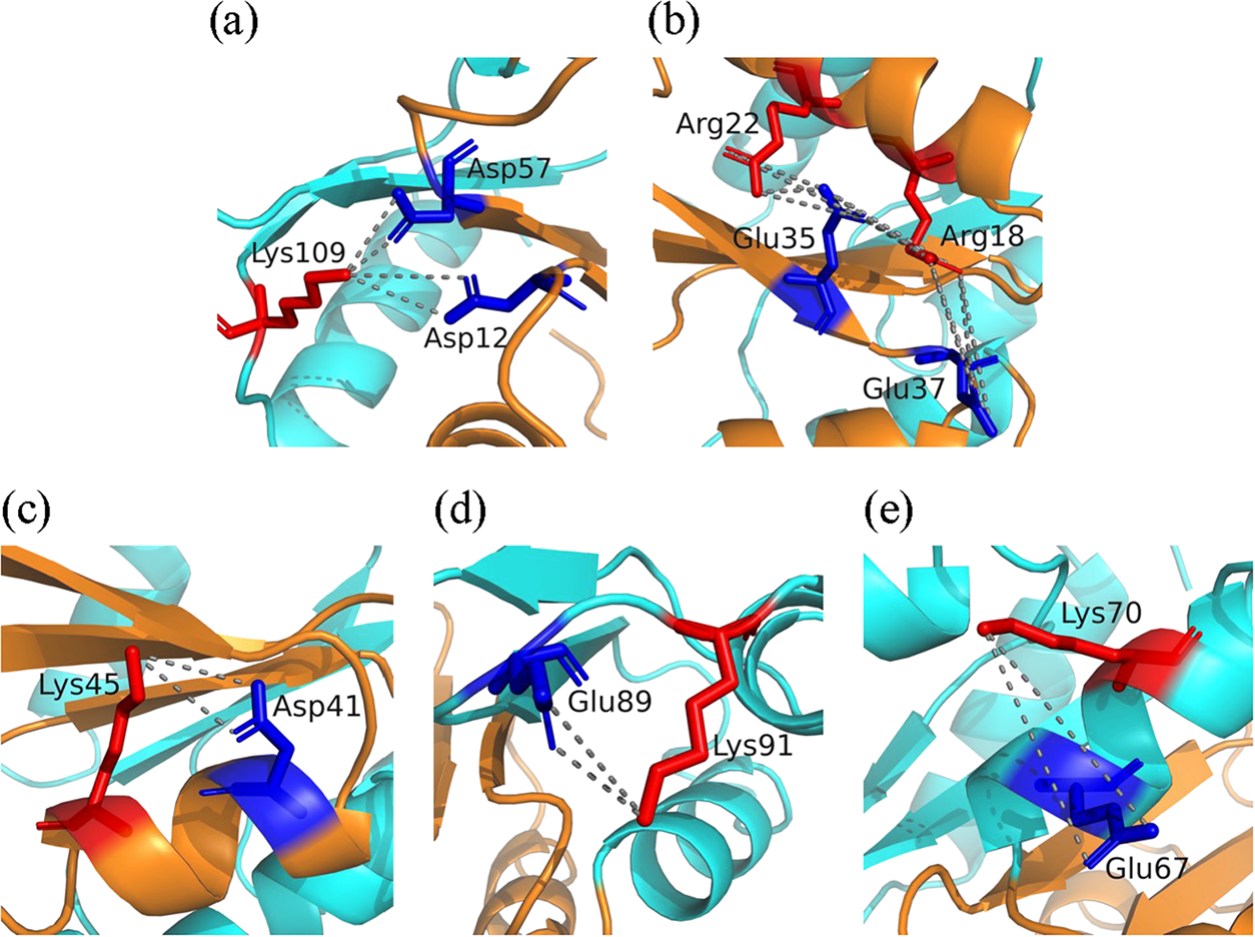
Spatial distribution of each salt bridge in the EcY protein: (a) the Asp12···Lys109···Asp57 triad; (b) the Arg22···Glu35···Arg18···Glu37 tetrad; (c) the Asp41···Lys45 dyad; (d) the Glu89···Lys91 dyad; and (e) the Glu67···Lys70 dyad. The N- and C-terminal domains are shown in orange and cyan, respectively. Acidic and basic residues participating in these interactions are highlighted in blue and red, respectively.

The triad involves the intermolecular salt bridges Asp12–Lys109 and Asp57–Lys109 and, therefore, is composed of the residues Asp12/Lys109/Asp57. Asp12 and Lys109 are located between the β_1_/α_1_ and β_5_/α_5_ loops, respectively, whereas Asp57 lies at the end of the β_3_-strand, linking the N- and C-terminal domains (Figure 11a). The Asp57–Lys109 salt bridge is stronger than the Asp12–Lys109 one: the latter begins to weaken at 374 K and is the most unstable of all observed salt bridges, while the former persists up to 450 K.

These findings are consistent with experimental evidence. Several studies have reported that the EcY active site is composed of Asp12, Asp13, Asp57, and Lys109; with Asp12 and Asp57 buried in the protein core, and Asp57 acting as the phosphorylation site.^9,10,38–42^ Because the three Asp residues cluster to form an acidic pocket at the edge of the β-sheet, leading to electrostatic repulsion at pH ≥ 5.1,^40^ there is a high probability to form salt bridges among these Asp residues and Lys109. Filimonov et al. and Volz and Matsumura reported only the Asp57-Lys109 salt bridge,^9,40^ and Volz and Matsumura concluded that this interaction is essential for understanding the consequences of phosphoryl-transfer reactions in EcY.^9^ In both analyses, two water molecules were observed in the active site, forming hydrogen bonds with Asp12, which likely hindered the detection of the Asp12-Lys109 salt bridge. However, Lee et al. reported that Asp12 forms a salt bridge with Lys109 in the crystal structure of activated EcY.^42^

While the triad links residues located mainly within disordered secondary structure elements of both domains, the tetrad connects residues situated mostly in ordered secondary structures of the N-terminal domain (Figure 11b). This tetrad is formed by residues Arg22/Glu35/Arg18/Glu37 through three intermolecular salt bridges: Arg22–Glu35, Arg18–Glu35, and Arg18–Glu37, all of which remain stable up to 400 K. The Arg22–Glu35 ion pair exhibits the highest stability; the Arg18–Glu35 pair increases its stability; and the Arg18–Glu37 interaction remains lowly stable between 302 and 400 K. However, all of these salt bridges lose stability at 450 K, significantly affecting the α_1_-helix, as two of its residues participate in these interactions. This observation is consistent with secondary structure analysis, which identifies the α_1_-helix as the most destabilized arrangement at this temperature. Moreover, this salt-bridge network aligns with the experimental evidence reported by Usher et al.,^11^ who identified the formation of five salt bridges in EcY, including interactions of Arg18 and Arg22 with Glu35 and Glu37.

The three intramolecular salt bridges form only dyads (Figure 11c–e). The Asp41–Lys45 ion pair, located in the N-terminal domain, is the most stable of all these interactions, maintaining nearly 70% of its interaction frequency up to 450 K. The other two pairs, situated in the C-terminal domain, are the weakest, with frequencies between 0.3 and 0.4 in the folded state. The Glu89–Lys91 pair exhibits frequencies below 0.3 from 374 K onward and is therefore considered negligible. In contrast, the Glu67–Lys70 pair fluctuates markedly with temperature: its frequency increases up to 374 K, decreases at 400 K, and rises again at 450 K. This behavior may reflect the spatial rearrangements undergone by the α_3_-helix.

These outcomes indicate that most of the secondary structures involved in the formation of salt bridges in EcY belong to the N-terminal domain. This arises because the residues forming the tetrad are located in the β_1_- and β_2_-strands and in the α_1_- and α_2_-helices; the two aspartic acid residues of the triad are positioned in the β_3_-strand and in the loop connecting β_1_ and α_1_; and the most stable dyad resides within the α_2_-helix. Specifically, the secondary structures responsible for salt bridge formation are (1) α_1_, α_2_, β_2_, β_3_, and the β_1_/α_1_ loop in the N-terminal domain, and (2) α_3_ and the β_4_/α_4_ and β_5_/α_5_ loops in the C-terminal domain. Consequently, salt bridges provide greater stability to the N-terminal domain. These observations align with the analyses of secondary structure and RMSF profiles, which show that the N-terminal domain is more stable than the C-terminal domain.

On the other hand, the TmY protein contains 15 salt bridges, of which 10 are intermolecular and five are intramolecular. With the exception of the Arg37–Glu68 ion pair, all salt bridges are maintained at frequencies greater than 0.3 from the folded to the unfolded state (Table 3), indicating that these interactions remain stable throughout the temperature range of 302 to 450 K. Figure 12 shows some spatial distribution of these salt bridges.

**Figure 12:**
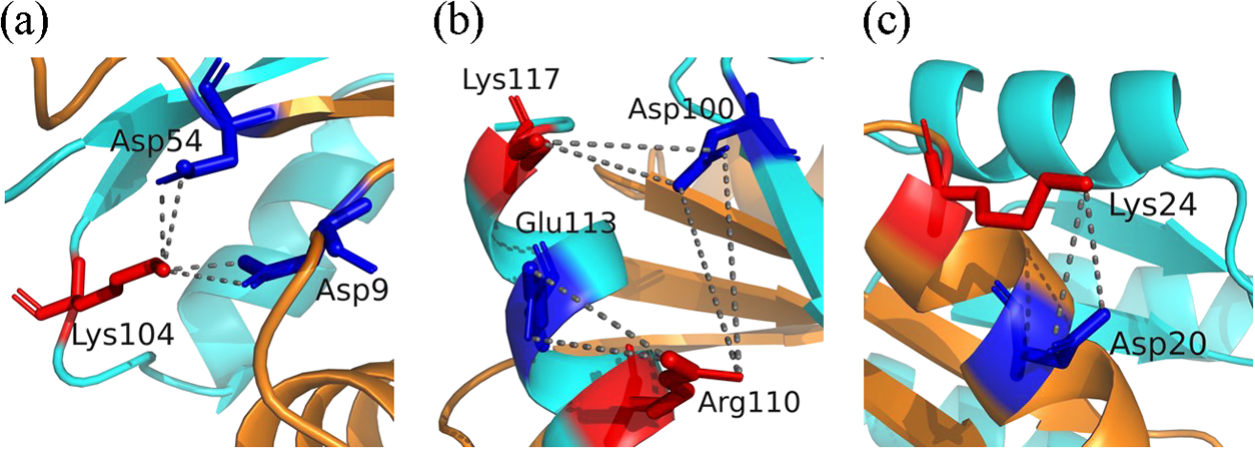
Spatial distribution of selected salt bridges in the TmY protein: (a) the Asp9···Lys104···Asp54 triad; (b) the Glu113···Arg110···Asp100···Lys117 tetrad; and (c) the Asp20···Lys24 dyad. The color scheme follows that in Figure 11. The complete set of salt bridges is provided in Figure S15 of the Supporting Information.

As shown, the thermophilic protein not only forms a larger number of salt bridges compared to the mesophilic protein but also establishes a greater number of salt-bridge networks. Specifically, two triads and two tetrads are identified. The triads are arranged as follows:

(a) Triad 1: Asp9···Lys104···Asp54, made up by the Asp9–Lys104 and Asp54–Lys104 salt bridges.
(b) Triad 2: Arg15···Glu32···Lys19, made up by the Arg15–Glu32 and Lys19–Glu32 salt bridges.

Meanwhile, the tetrads are composed of

(a) Tetrad 1: Asp64···Arg37···Glu68···Lys71, made up by the Arg37–Asp64, Arg37–Glu68, and Glu68–Lys71 salt bridges.
(b) Tetrad 2: Glu113···Arg110···Asp100···Lys117, made up by the Arg110–Glu113, Asp100–Lys117, and Asp100–Arg110 salt bridges.

Triad 1 comprises the strongest salt bridges identified in this study and connects two secondary structures of the N-terminal domain (the loop located between β_1_/α_1_ and β_3_ strand) with the final loop situated between β_5_/α_5_ in the C-terminal domain (Figure 12a). Both ionic pairs maintain highly stable interactions up to 400 K, with formation frequencies ranging from 94 to 98%. Although the Asp54–Lys104 salt bridge weakens slightly at 450 K, it still exhibits high stability, forming on average 88.5% of the time. In contrast, the Asp9–Lys104 interaction decreases by nearly 50% between 400 and 450 K, reaching a frequency of 0.486, consistent with stability near the moderate level. These results align with previous molecular dynamics simulations performed over shorter trajectories of 10 and 20 ns^43^ and with experimental evidence reported by Usher et al.,^11^ who demonstrated that metal binding does not disrupt the salt bridge formed between the conserved Lys104 and the active-site residue Asp54.

As can be inferred from these results, this salt-bridge triad is conserved in both proteins, being formed by Asp12···Lys109···Asp57 in EcY and Asp9···Lys104···Asp54 in TmY. Despite the sequence offsets, both triads preserve the same spatial architecture and connect homologous structural regions, possibly indicating a conserved functional role. This conclusion is further reinforced by a multiple sequence alignment of CheY proteins from *T. maritima*, *E. coli*, *B. subtilis*, *Salmonella typhimurium*, *Rhizobium meliloti*, and *Halobacterium salinarum*, which reveals strict conservation of the Asp-Lys-Asp motif across these organisms. This alignment was performed using the Clustal Omega program (https://www.ebi.ac.uk/jdispatcher/msa), and the corresponding alignment highlighting the conserved residues is provided in Figure S14 of the Supporting Information.

Importantly, this triad involves the active-site aspartate, Asp57 in EcY and Asp54 in TmY, which acts as the phosphorylation site during CheY activation.^9–11,38–42^ In this context, the Asp residue serves as an electrostatic anchor for phosphate binding, triggering the conformational transition that enables downstream flagellar signaling. The conservation of this triad across species with markedly different optimal growth temperatures highlights its fundamental biological relevance, extending beyond purely local thermodynamic stabilization.

Although direct experimental evidence for the simultaneous formation of the complete triad remains limited, the literature data^9,11,40,42^ combined with our molecular dynamics results support a plausible functional scenario. Upon phosphorylation of the active-site Asp (Asp57 or Asp54), the electrostatic interaction between this residue and the C-terminal Lys (Lys109 in EcY or Lys104 in TmY) is likely weakened or disrupted due to charge redistribution. Under these conditions, the Lys residue may preferentially engage with the N-terminal Asp (Asp12 or Asp9), thereby preserving a stabilizing electrostatic interaction between the N- and C-terminal domains. This potential dynamic reorganization of interactions in the triad may provide a mechanistic link among phosphorylation, structural rearrangements, and interdomain communication. Therefore, the Asp–Lys–Asp salt-bridge network may function as a switching motif rather than as a persistent stabilizing scaffold in CheY proteins.

These observations suggest that future molecular dynamics studies explicitly incorporating the phosphorylated state of CheY would be particularly valuable to further elucidate how activation-related electrostatic rearrangements are coupled to thermal stability in both thermophilic and mesophilic proteins.

The residues of Triad 2 are located within secondary structures of the N-terminal domain (α_1_-helix and β_2_ strand), and both salt bridges maintain moderate stability (61.8–71.5%) across the 302–450 K range. Consequently, this network contributes to maintaining the stability of this domain.

Tetrad 1 contains salt bridges with distinct spatial arrangements. The Arg37–Glu68 and Arg37-Asp64 ion pairs are intermolecular, both linking the α_2_- and α_3_-helices located in the N- and C-terminal domains, respectively. In contrast, the Glu68–Lys71 ion pair is intramolecular and lies within the α_3_-helix of the C-terminal domain. The frequencies of these interactions display opposite trends as the temperature increases from 302 to 450 K: for instance, the Arg37–Glu68 salt bridge decreases from low stability to negligible, whereas the Glu68–Lys71 interaction increases from low to moderate stability. This contrasting behavior arises because Arg37 and Glu68 undergo structural rearrangements that separate them, weakening their interactions, while Glu68 and Lys71, being part of the same helix, preferentially interact with each other.

The three salt bridges forming Tetrad 2 are located exclusively within the secondary structures of the C-terminal domain (β_5_-strand and α_5_-helix) (Figure 12b) and exhibit moderate-to-low stability in the temperature range of 302 to 450 K. Among them, the intramolecular Arg110–Glu113 salt bridge is the least stable, with its frequency decreasing from moderate to low as temperature increases. The Asp100–Lys117 salt bridge retains moderate stability with nearly constant frequencies, ranging between 0.524 and 0.630. In contrast, the Asp100–Arg110 interaction strengthens from 302 to 400 K, reaching *f*_SB_ = 0.741 (approaching the high-stability threshold) but decreases at 450 K, showing *f*_SB_ = 0.570.

In addition to these triads and tetrads, which together comprise 10 salt bridges, five additional salt bridges form simple dyads: two intermolecular and three intramolecular. The residues participating in these dyads are distributed across five secondary structures in the N-terminal domain and three in the C-terminal domain.

These dyads exhibit different trends as the temperature increases; i.e., their behaviors are not uniform, as some interactions weaken while others become more stable. For example: (1) the Asp20–Lys24 dyad, located in the α_1_-helix (Figures 12c), is the most stable among them but gradually loses interaction strength with increasing temperature, showing average frequencies of 0.909 at 302 K and 0.849 at 400 K, representing a 6% decrease in interaction, and then dropping to *f*_SB_ = 0.740 at 450 K; (2) the Glu41–Lys44 pair, located in the α_2_-helix, increases in stability, rising from an average frequency of 0.362 at 302 to 0.463 at 450 K; and (3) the Arg4–Glu28 salt bridge, situated between the β_1_- and β_2_-strands, maintains an approximately constant frequency of ∼0.425 across the entire temperature range (302–450 K).

Nearly all secondary structures of TmY participate two or more times in the formation of salt bridges, either directly or through adjacent disordered regions, thereby reinforcing the structural robustness of the protein. The only exception is helix α_4_-helix, which participates solely in the intramolecular Glu92–Lys95 salt bridge. The frequency of this interaction increases from 302 K (*f*_SB_ = 0.427) to 400 K (*f*_SB_ = 0.552) but subsequently decreases at 450 K (*f*_SB_ = 0.346), indicating destabilization at high temperatures, which is consistent with the loss of structural integrity previously noted in the secondary structure analysis. Additionally, the β_4_-strand is the only ordered secondary structure that does not participate in salt bridge formation, as it lacks charged residues and instead contains three hydrophobic ones. Consequently, aside from this strand, all ordered secondary structures and three loops engage in one or more salt bridges, collectively contributing to the high structural stability of the protein. These observations reveal a consistent pattern in which salt bridge formation broadly stabilizes the TmY fold.

Furthermore, as the protein undergoes structural rearrangements with increasing temperature, new non-native salt bridges can emerge, namely, interactions that exhibit average frequencies greater than 0.3 at temperatures above 302 K. In the EcY protein, two salt bridges with low stability are detected: (1) Glu34–Lys7, with *f*_SB_ = 0.309 at 400 K and *f*_SB_ = 0.337 at 450 K; and (2) Arg19–Asp12, with *f*_SB_ = 0.311 at 450 K. In the TmY protein, three salt bridges are formed, including one that increases from low to moderate stability and two that persist at low stability: (1) Asp64–Lys67, with *f*_SB_ = 0.368 at 374 K, *f*_SB_ = 0.392 at 400 K, and *f*_SB_ = 0.510 at 450 K; (2) Arg37–Glu59, with *f*_SB_ = 0.431 at 450 K; and (3) Glu38–Lys42, with *f*_SB_ = 0.316 at 450 K.

These findings indicate that, beyond possessing a greater number of salt bridges and salt-bridge networks than the EcY protein, the TmY protein also exhibits an enhanced capacity to form additional interactions at elevated temperatures. As TmY remains compact and structurally stable, temperature-induced rearrangements bring oppositely charged residues into closer proximity, thereby facilitating the formation of new electrostatic contacts.

Finally, in the experimental work of Usher et al.,^11^ the formation of five salt bridges was stated in both proteins. They concluded that the thermal stability of the TmY protein arises from many small, likely additive, stabilizing differences relative to its mesophilic homologues, among which EcY is included. However, in our study, we observed that TmY contains a greater number of salt bridges and salt-bridge networks compared with EcY. Moreover, the ionic pairs connect a larger set of secondary structures and terminal-domain regions, spreading throughout the entire TmY structure. This contrasts with that of EcY, whose salt bridges are mainly confined to the N-terminal domain. Although Usher and collaborators found an equal number of salt bridges in both proteins and their conclusion remains valid, we argue that the thermal stability of TmY is not merely the result of small additive stabilizing effects but rather to a strong contribution from salt bridges that enables TmY to withstand temperature changes. Therefore, the cooperativity among salt bridges is key for TmY to maintain a stable and compact structure at high temperatures.

These arguments are further supported by preliminary mutational analyses in which key salt-bridge–forming residues in TmY, namely, Arg4, Arg15, Glu32, Asp49, and Lys104, were individually substituted. Although the detailed results of these point mutations will be reported in a subsequent study, these initial observations reinforce the essential contribution of specific ionic interactions to the thermal resilience of the protein. In the present work, our primary objective has been conducted to compare the structural behavior of both proteins and to identify the interactions that allow TmY to endure temperature increases more effectively than its mesophilic counterpart. Through this comparative analysis, we highlight not only the enhanced stability of the individual salt bridges and salt-bridge networks in TmY but also the role of the secondary structural elements in positioning charged residues in close proximity, thereby facilitating the formation and persistence of these interactions. Collectively, these findings underscore the cooperative nature of the stabilizing forces that enable TmY to maintain a compact and robust fold at elevated temperatures.

In summary, although hydrogen bonds and salt bridges have long been key contributors to thermophilic protein stability, the present study provides new insight into how these interactions operate cooperatively under dynamic and temperature-dependent conditions. By combining microsecond-scale MD simulations with a residue- and structure-resolved analysis, we show that thermal resilience is not governed by the mere presence of stabilizing interactions but by their persistence, structural coupling, and integration within compact protein architectures. Our results indicate that hydrogen-bond networks alone are insufficient to maintain structural integrity at elevated temperatures, as exemplified by EcY, where a lower number and reduced persistence of salt bridges lead to progressive loss of compactness and unfolding despite the presence of hydrogen bonds. In contrast, the exceptional thermal stability of TmY critically depends on the presence of persistent salt bridges that act as structural anchors or molecular staples, reinforcing hydrogen-bond networks and preserving a compact fold across the entire temperature range studied. This comparative framework reveals mechanistic differences between thermophilic and mesophilic proteins that cannot be inferred from static structure alone and highlights the essential role of salt-bridge-mediated cooperativity in enabling protein stability under extreme thermal conditions.

## 4 Conclusions

Although the CheY protein from *T. maritima* had been examined in previous studies, important physicochemical details regarding the specific interactions that contribute to its exceptional thermal stability had remained insufficiently characterized. In this molecular dynamics work, the results indicate that salt bridges and their organization in cooperative networks play an important stabilizing mechanism that preserves the structural integrity of TmY under thermal stress. In comparison with its mesophilic homologue from *E. coli*, TmY tends to form a greater number of ionic interactions, some of which are organized into extended networks that connect multiple secondary-structure elements and couple the N- and C-terminal domains. These electrostatic patterns span the entire protein fold and act as a cohesive stabilizing scaffold capable of limiting local flexibility, preserving global compactness, and favoring the maintenance of native topological contacts even at elevated temperatures.

From a mechanistic and biological standpoint, this distributed electrostatic framework may represent a strategy that contributes to thermophilic adaptation, allowing TmY to maintain its native fold and domain communication under extreme thermal conditions. By stabilizing both local secondary structures and long-range interdomain contacts, salt-bridge networks appear to support the idea that the protein remains structurally competent at high temperatures. Therefore, structural reinforcement, driven by the cooperativity among multiple salt bridges, offers a plausible explanation for the remarkable thermostability of TmY.

To further strengthen these findings, targeted point mutations of key residues involved in the most stabilizing salt bridges have been initiated, and a detailed analysis of their effects will be presented in a future study.

## Supplementary Material





## Data Availability

All programs and data used in this manuscript, including the scripts with the appropriate parameters (.mdp files), topology files (topol.top), position-restraint files for heavy atoms (posre.itp), structural coordinate files (.pdb and .gro), and the Python script for the analysis of secondary structures (SS_analysis.py) are available in Data_Files set. In addition, the most important output files generated, such as energy minimization logs (em.log), initial production configurations (conf0.pdb), and the complete 1 μs production logs (md_1 ms.log), are also included. A README file is also provided to describe the contents and organization of these data (Data_Files.zip).

## Notes

Data files (ZIP)
Structural analyses: Root-mean-square deviation at *L* = 1.0 nm and *L* = 2.0 nm (Figure S1); radius of gyration at *L* = 1.0 nm and *L* = 2.0 nm (Figure S2); root-mean-square deviation (Figure S3); radius of gyration (Figure S4); fraction of native contacts (Figure S5); TmY and EcY conformations (Figure S6); ILV clusters (Figure S7); trends of each α-helix (Figure S8); trends of each β-strand (Figure S9); secondary structure profiles (Figures S10, S11, and S12); root-mean-square fluctuation (Figure S13); root-mean-square deviation (Table S1), radius of gyration (Table S2); fraction of native contacts (Table S3); total percentage of α-helices (Table S4); percentages of α_1_-, α_2_-, α_3_-, α_4_-, α_5_-, and 3_10_-helices (Table S5); total percentage of β-strands (Table S6); percentages of β_1_-, β_2_-, β_3_-, β_4_-, and β_5_-strands (Table S7); molecular interaction analyses: Multiple sequence alignment (Figure S14); salt bridges in TmY (Figure S15); number of HBpp (Table S8); and number of HBps (Table S9) (PDF)
