# Supplementary material for: Comparative Molecular Dynamics Study of the Thermal Stability of CheY Proteins from Hyperthermophilic and Mesophilic Organisms
Source: J Chem Inf Model. 2026 Mar 10;66(6):3259–75. doi: 10.1021/acs.jcim.5c02944 (PMC13014450; doi:10.1021/acs.jcim.5c02944)
Supplement: Supplementary file 2 [file ci5c02944_si_002.pdf]

Supporting Information

Comparative Molecular Dynamics Study of the Thermal Stability of CheY Proteins from Hyperthermophilic and Mesophilic Organisms

Salomón J. Alas-Guardado,*,†,‡ Melisa S. Anzures-Mendoza,† José Y. Sol-Fragoso,† and Edgar López-Pérez§

† Departamento de Ciencias Naturales, Universidad Autónoma Metropolitana Unidad Cuajimalpa, Ciudad de México 05348, México
‡ Departamento de Química, Universidad Autónoma Metropolitana Unidad Iztapalapa, Ciudad de México 09310, México
§ Department of Molecular & Cell Biology, University of Connecticut, Storrs, Connecticut 06269, United States

* Email: salas@cua.uam.mx

This Supporting Information provides complementary structural and molecular interaction analyses that extend and support the results discussed in the main manuscript. It includes detailed evaluations of global and local structural stability descriptors (RMSD, Rg, Q, RMSF, and SS content), representative protein conformations, and illustrative hydrophobic ILV cluster. In addition, it presents the average hydrogen bond values, a multiple sequence alignment highlighting conserved residues among CheY homologues, and a detailed visualization of salt bridges in TmY. Comprehensive tabulated data summarizing the metrics of RMSD, Rg, Q, SS content, and HB are provided. Together, these data offer additional mechanistic insight into the thermal stability and structural resilience of CheY proteins across different temperature regimes. This Supporting Information is organized as follows:

Structural analyses

1. Root-mean-square deviation at L = 1.0 nm and L = 2.0 nm (Figure S1)
2. Radius of gyration at L = 1.0 nm and L = 2.0 nm (Figure S2)
3. Root-mean-square deviation (Figure S3)
4. Radius of gyration (Figure S4)
5. Fraction of native contacts (Figure S5)
6. Tables: Root-mean-square deviation (Table S1); Radius of gyration (Table S2); Fraction of native contacts (Table S3)
7. TmY and EcY conformations (Figure S6)
8. ILV clusters (Figure S7)

9. Tables: Total percentage of α-helices (Table S4); Percentages of α_1-, α_2-, α_3-, α_4-, α_5-, and 3_{10}-helices (Table S5)
10. Trends of each α-helix (Figure S8)
11. Tables: Total percentage of β-strands (Table S6); Percentages of β_1-, β_2-, β_3-, β_4-, and β_5-strands (Table S7)
12. Trends of each β-strand (Figure S9)
13. Secondary structure profiles (Figures S10, S11, and S12)
14. Root-mean-square fluctuation (Figure S13)

Molecular interaction analyses

15. Tables: Number of HBpp (Table S8); Number of HBps (Table S9)
16. Multiple sequence alignment (Figure S14)
17. Salt bridges in TmY (Figure S15)

Structural analyses

1. Root-mean-square deviation at L = 1.0 nm and L = 2.0 nm

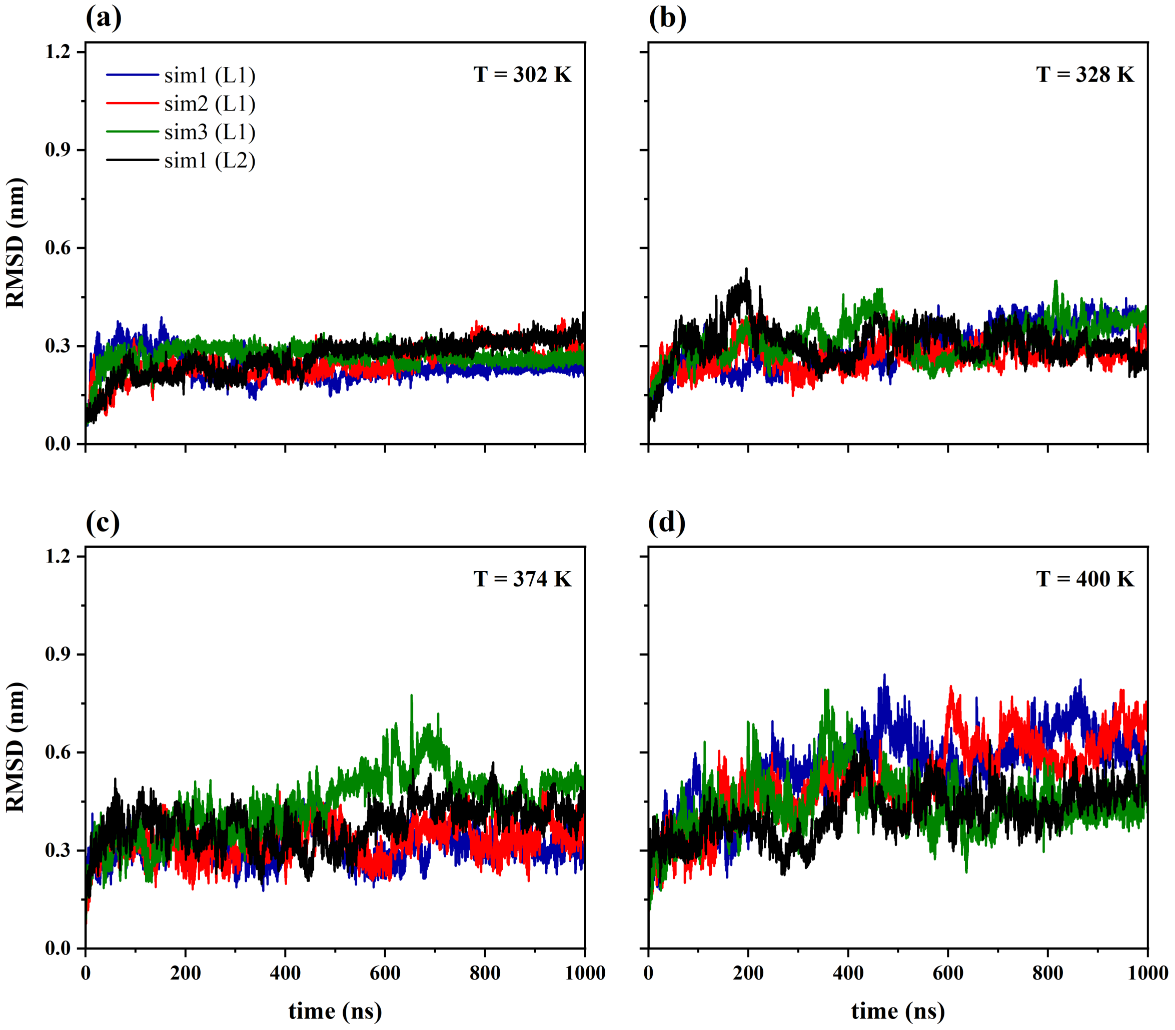

Figure S1. Time series of the three independent RMSD trajectories (replicas) for the EcY protein at 302, 328, 374, and 400 K. The blue, red, and green lines correspond to replicas 1, 2, and 3, respectively, simulated at L = 1.0 nm, whereas the black lines represent simulations performed at L = 2.0 nm. The labels L1 and L2 indicate systems with distances of L = 1.0 nm and L = 2.0 nm, respectively.

2. Radius of gyration at L = 1.0 nm and L = 2.0 nm

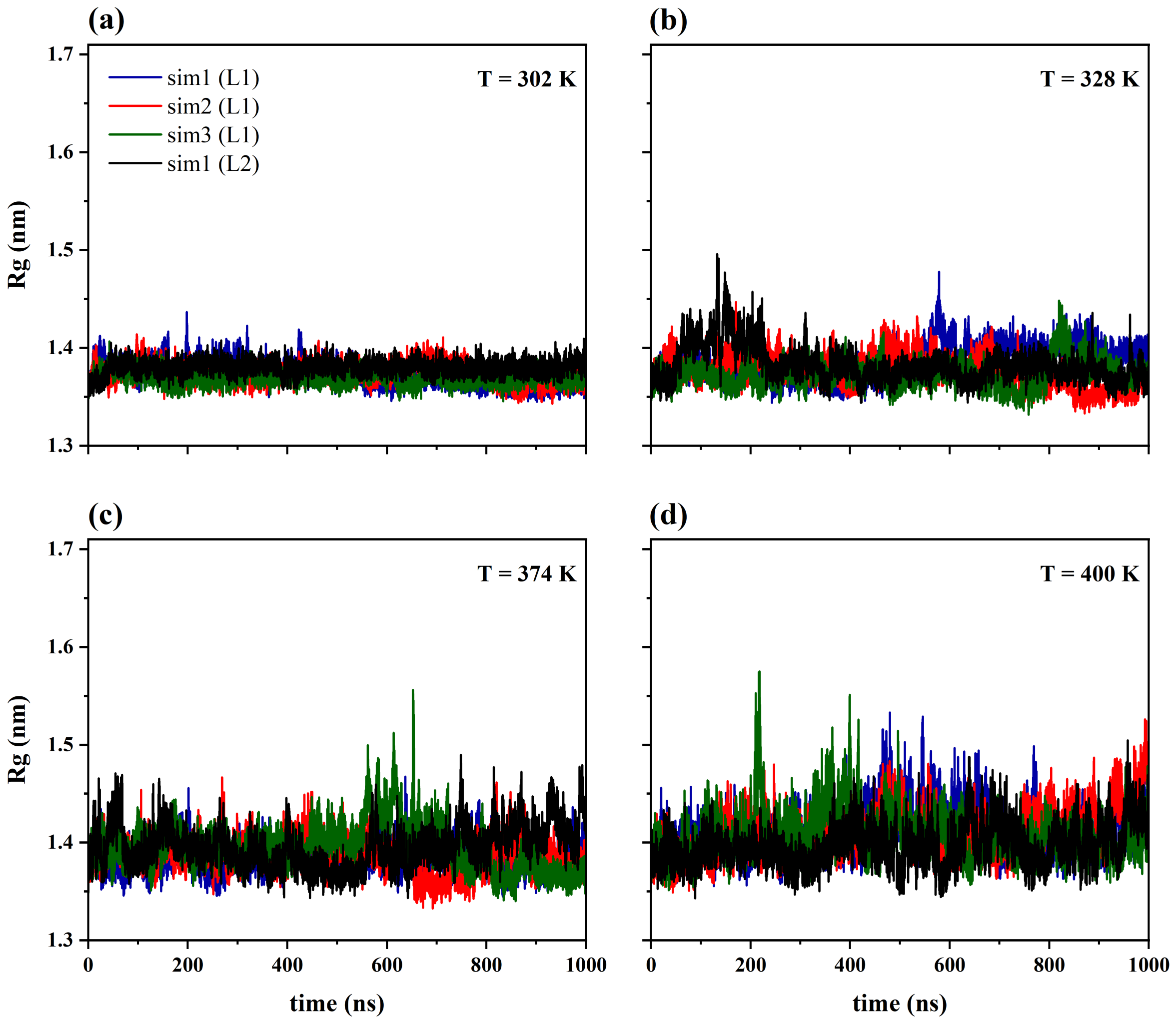

Figure S2. Time series of the three independent Rg trajectories (replicas) for the EcY protein at 302, 328, 374, and 400 K. The blue, red, and green lines correspond to replicas 1, 2, and 3, respectively, simulated at L = 1.0 nm, whereas the black lines represent simulations performed at L = 2.0 nm. The labels L1 and L2 indicate systems with distances of L = 1.0 nm and L = 2.0 nm, respectively.

3. Root-mean-square deviation

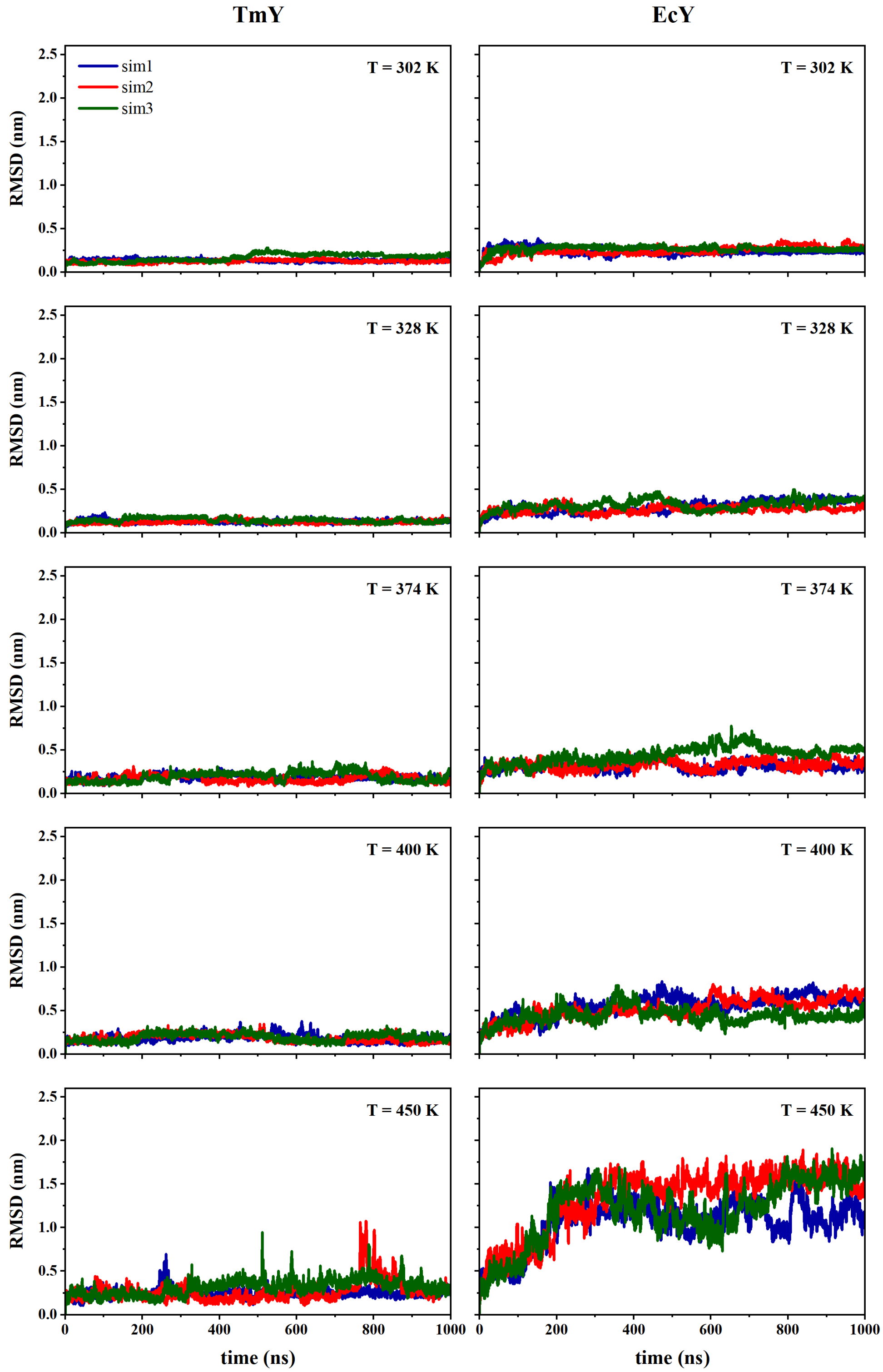

Figure S3. RMSD trajectories for the TmY and EcY proteins at 302, 328, 374, 400, and 450 K. The blue, red, and green lines indicate the simulations or replicas 1, 2, and 3, respectively.

4. Radius of gyration

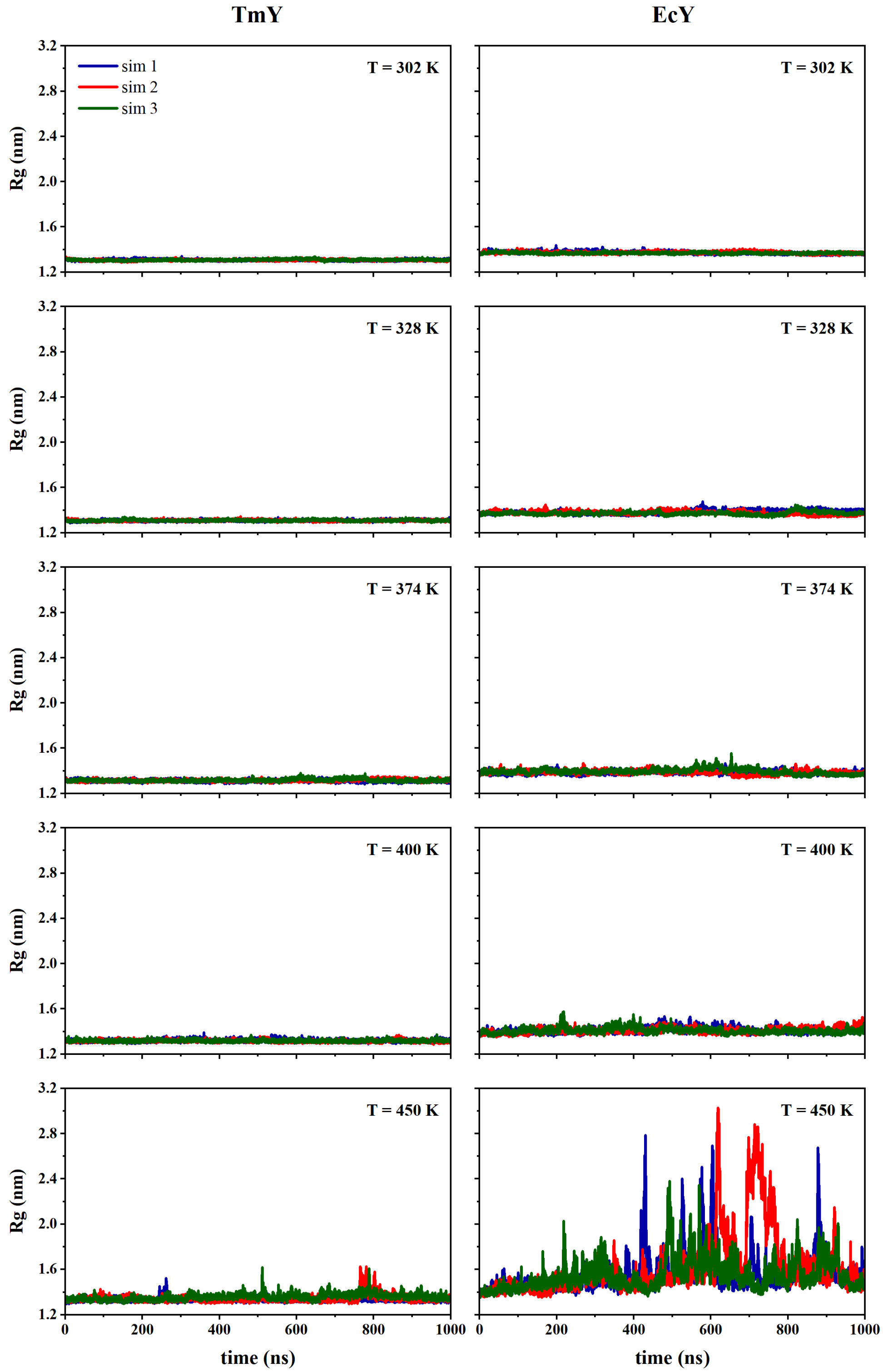

Figure S4. Rg trajectories for the TmY and EcY proteins at 302, 328, 374, 400, and 450 K. The blue, red, and green lines indicate the simulations or replicas 1, 2, and 3, respectively.

5. Fraction of native contacts

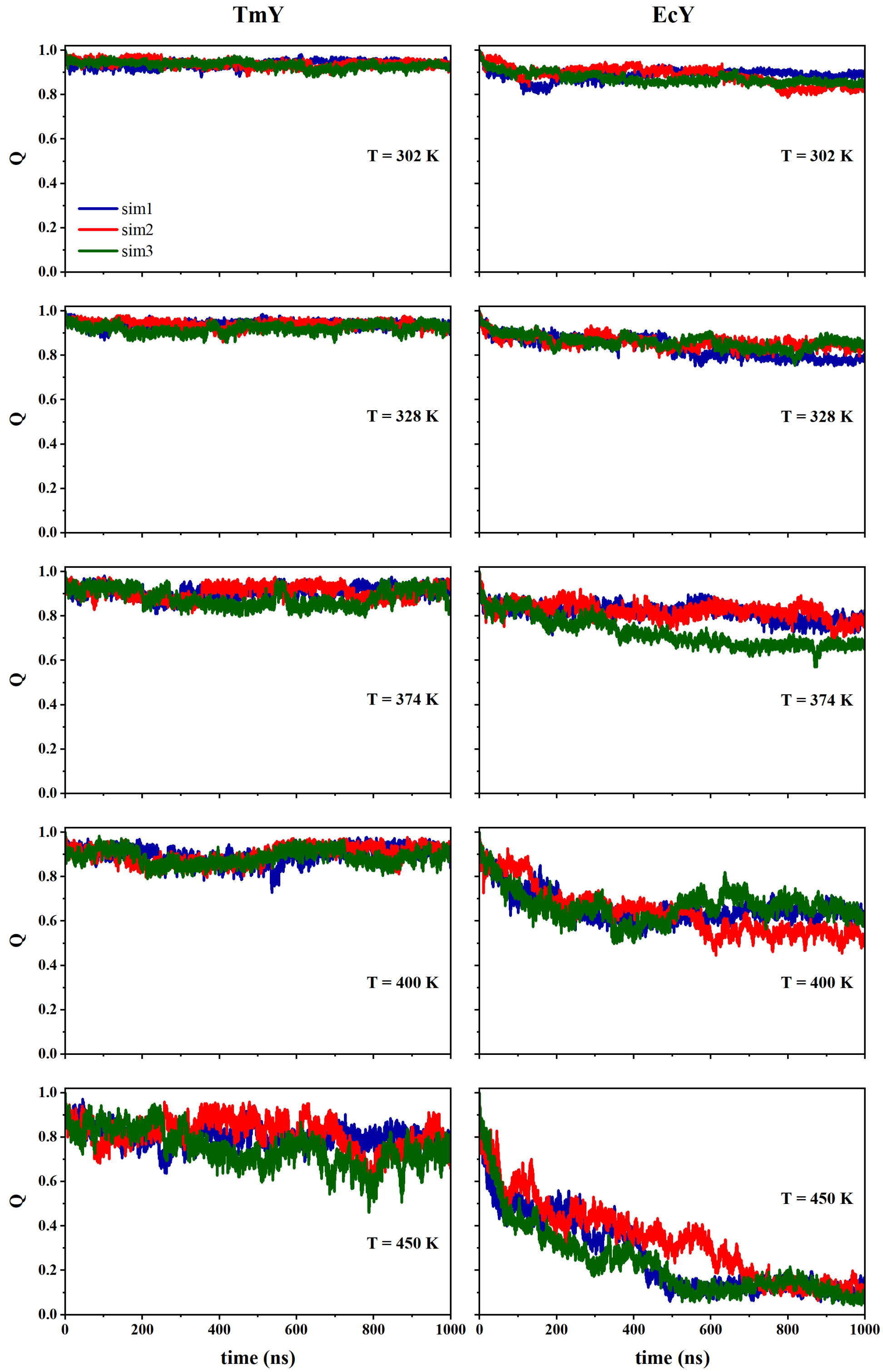

Figure S5. Q trajectories for the TmY and EcY proteins at 302, 328, 374, 400, and 450 K. The blue, red, and green lines indicate the simulations or replicas 1, 2, and 3, respectively.

6. **Tables:** Statistical descriptors of the RMSD, Rg, and Q structural parameters for the TmY and EcY proteins obtained from three MD simulation replicas at the five analyzed temperatures; μ = mean, σ = standard deviation, and m = median.

Table S1. Root-mean-square deviation.

T (K)	TmY			EcY		
	RMSD (nm)					
	μ	σ	m	μ	σ	m
302	0.131	± 0.017	0.131	0.250	± 0.043	0.250
328	0.134	± 0.022	0.131	0.295	± 0.061	0.288
374	0.181	± 0.044	0.177	0.361	± 0.090	0.344
400	0.184	± 0.042	0.180	0.502	± 0.120	0.499
450	0.273	± 0.093	0.250	1.193	± 0.347	1.238

Table S2. Radius of gyration.

T (K)	TmY			EcY		
	Rg (nm)					
	μ	σ	m	μ	σ	m
302	1.308	± 0.006	1.308	1.372	± 0.010	1.371
328	1.309	± 0.007	1.309	1.379	± 0.017	1.377
374	1.315	± 0.010	1.314	1.388	± 0.019	1.387
400	1.319	± 0.010	1.318	1.409	± 0.024	1.405
450	1.344	± 0.027	1.338	1.523	± 0.089	1.505

Table S3. Fraction of native contacts.

T (K)	TmY			EcY		
	Q					
	μ	σ	m	μ	σ	m
302	0.939	± 0.016	0.940	0.884	± 0.031	0.886
328	0.935	± 0.019	0.938	0.854	± 0.038	0.857
374	0.902	± 0.035	0.908	0.789	± 0.066	0.809
400	0.898	± 0.036	0.902	0.657	± 0.084	0.648
450	0.792	± 0.073	0.798	0.311	± 0.149	0.303

7. TmY and EcY conformations

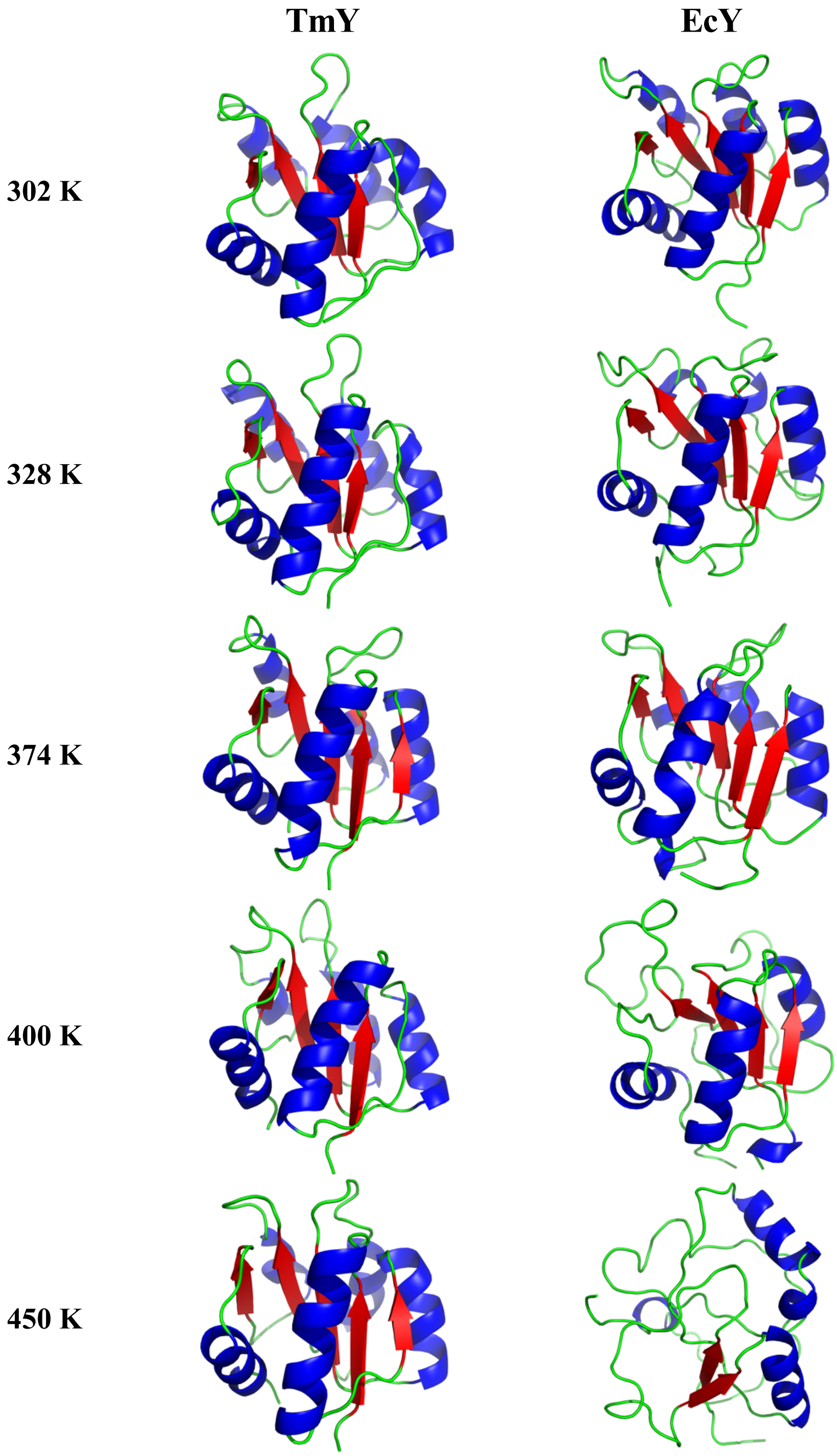

Figure S6. Representative structural snapshots showing the temperature-dependent evolution of the TmY and EcY proteins. Each snapshot corresponds to the final conformation (t = 1,000 ns) obtained from the first independent MD trajectory at each simulation temperature.

8. ILV clusters

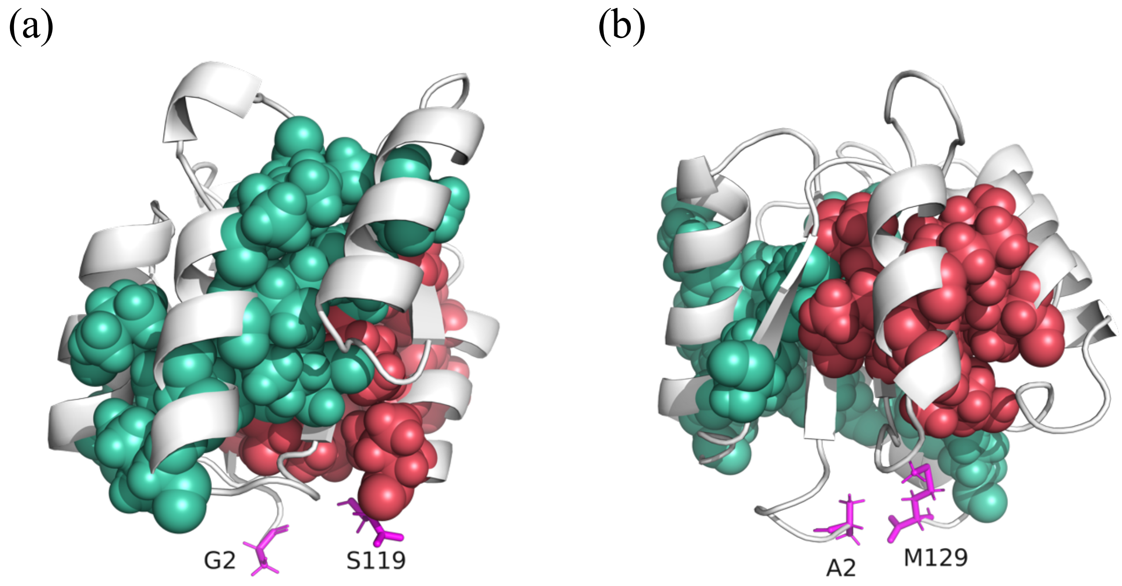

Figure S7. ILV clusters in (a) TmY and (b) EcY proteins at 302 K from the first configuration (*t* = 0 ns) of simulation 1. The main cluster (cluster 0) is shown in red spheres, while cluster 1 is represented by aqua-green spheres. The N- and C-terminal residues are highlighted in magenta.

9. **Tables:** Statistical descriptors of the α-helices for the TmY and EcY proteins obtained from three MD simulation replicas at the five analyzed temperatures; μ = mean, σ = standard deviation, and m = median.

Table S4. Total percentage of α-helices.

T (K)	TmY			EcY		
	α-helix (%)					
	μ	σ	m	μ	σ	m
302	40.129	± 3.104	39.831	32.167	± 3.222	32.031
328	39.858	± 4.310	40.678	31.050	± 3.746	31.250
374	36.608	± 4.466	36.441	27.789	± 6.017	28.906
400	35.976	± 4.692	36.441	20.084	± 6.032	19.531
450	27.532	± 6.674	27.966	10.668	± 5.270	10.156

Table S5. Percentages of α_1-, α_2-, α_3-, α_4-, α_5-, and 3_{10}-helices.

T (K)	TmY		EcY	
	α_1-helix (%)			
	μ	σ	μ	σ
302	10.498	0.634	9.871	0.631
328	10.390	0.656	9.878	0.860
374	10.292	0.768	7.734	2.605
400	10.317	1.033	6.177	2.797
450	8.210	2.816	2.247	2.484

TmY		EcY	
α_2-helix (%)			
μ	σ	μ	σ
7.669	1.564	6.015	0.548
8.133	1.658	5.970	0.591
8.021	1.709	5.862	0.744
7.975	1.767	5.605	1.026
6.810	2.297	4.037	2.353

T (K)	TmY		EcY	
	α_3-helix (%)			
	μ	σ	μ	σ
302	7.556	1.537	5.575	0.856
328	6.876	2.420	5.317	1.647
374	4.881	3.150	4.861	1.993
400	4.767	3.164	2.542	2.466
450	3.259	2.820	2.071	2.167

TmY		EcY	
α_4-helix (%)			
μ	σ	μ	σ
6.408	1.380	3.951	2.467
6.573	1.572	3.511	2.337
5.623	1.878	3.576	2.616
5.332	2.385	1.079	1.895
2.281	2.569	0.562	1.355

T (K)	TmY		EcY	
	α_5-helix (%)			
	μ	σ	μ	σ
302	7.640	1.057	6.755	1.979
328	7.591	1.089	6.374	1.910
374	7.458	1.251	5.758	1.914
400	7.261	1.201	4.680	2.814
450	6.223	2.396	1.751	2.327

TmY	
3_{10}-helix (%)	
μ	σ
0.358	0.884
0.295	0.815
0.332	0.846
0.324	0.826
0.751	0.988

10. Trends of each α-helix of the TmY and EcY proteins from three MD simulation replicas at the five analyzed temperatures.

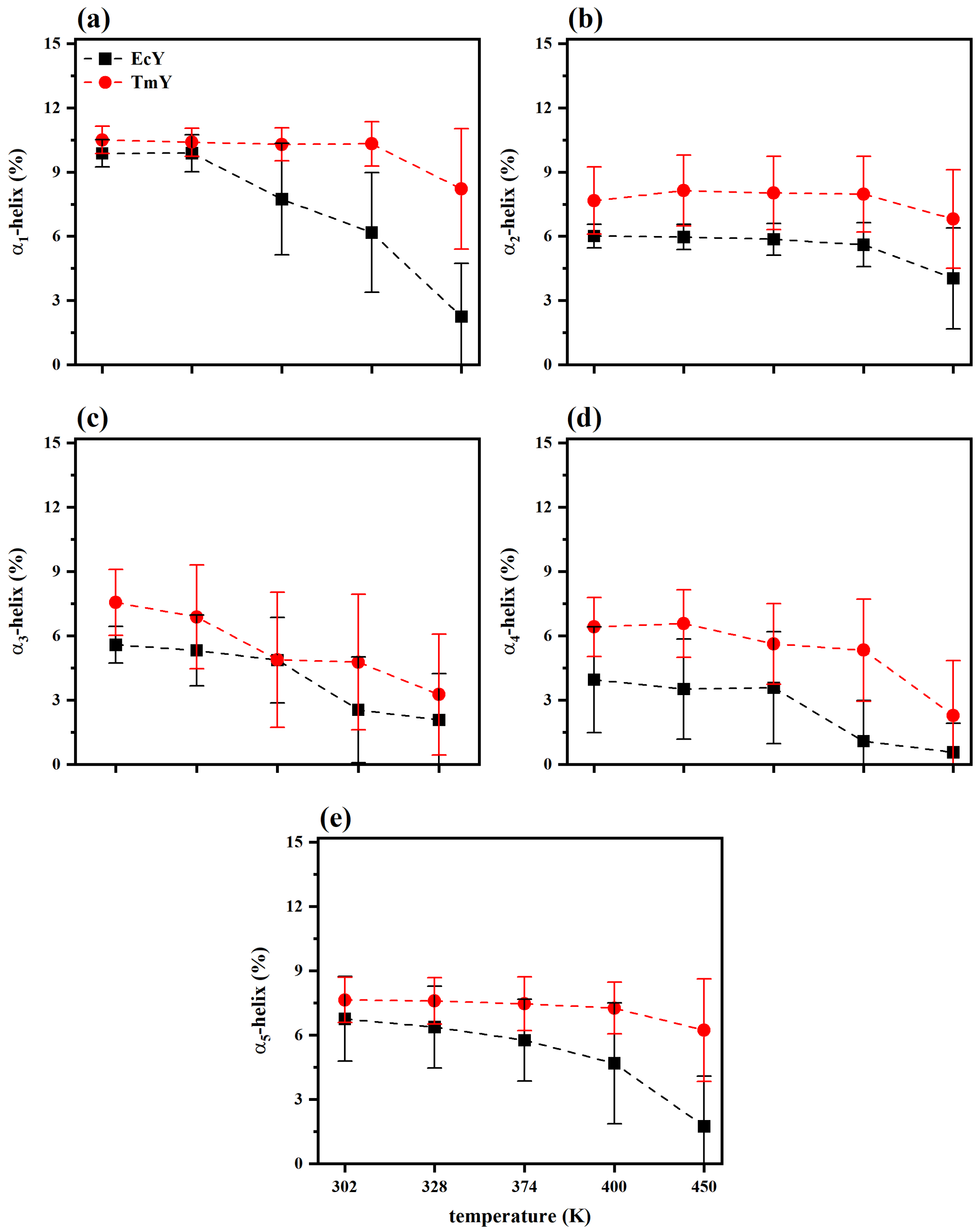

Figure S8. Average percentages and standard deviation values of (a) α_1-, (b) α_2-, (c) α_3-, (d) α_4-, (e) α_5-helices. Symbols represent the mean values, and error bars indicate the standard deviations. Dashed lines are shown as guides for the eye.

Description of α-helix percentages

The α-helix content of the mesophilic protein is lower than that of the thermophilic counterpart at 302 K, that is, TmY and EcY proteins contain 40.13 ± 3.10% and 32.17 ± 3.22% elements, respectively. This trend is maintained across all temperatures. For instance, at 450 K, the TmY protein exhibits 27.53 ± 6.67%, whereas the EcY protein shows 10.67 ± 5.27%. In other words, both proteins lose on average 12.60 and 21.50% of their α-helical content from the folded to the unfolded state, respectively.

Both proteins contain five α-helices whose content decreases as the temperature increases. The mesophilic EcY protein loses a greater amount of α_1- and α_5-helical structures than its thermophilic homologue TmY over the temperature range from 302 to 450 K; EcY loses on average 7.62 and 5.00%, whereas TmY loses 2.29 and 1.42%, respectively. In contrast, the TmY protein exhibits a slightly larger loss in α_3- and α_4-helices compared to EcY within the same temperature interval, with TmY decreasing by 4.30 and 4.13%, and EcY by 3.50 and 3.39%, respectively.

11. **Tables:** Statistical descriptors of the β-strands for the TmY and EcY proteins obtained from three MD simulation replicas at the five analyzed temperatures; μ = mean, σ = standard deviation, and m = median.

Table S6. Total percentage of β-strands.

T (K)	TmY			EcY		
	β strand (%)					
	μ	σ	m	μ	σ	m
302	20.855	± 1.309	21.186	16.976	± 0.950	16.406
328	20.761	± 1.215	21.186	16.776	± 0.561	17.188
374	20.544	± 1.576	20.339	16.389	± 0.855	16.406
400	20.495	± 1.580	21.186	15.469	± 1.625	15.625
450	19.660	± 2.259	20.339	7.164	± 4.816	7.813

Table S7. Percentages of β_1-, β_2-, β_3-, β_4-, and β_5-strands.

T (K)	TmY		EcY	
	β_1-strand (%)			
	μ	σ	μ	σ
302	4.154	0.273	3.233	0.274
328	4.168	0.243	3.122	0.054
374	4.143	0.288	3.110	0.115
400	4.140	0.305	3.043	0.259
450	4.073	0.459	2.050	1.246

TmY		EcY	
β_2-strand (%)			
μ	σ	μ	σ
4.850	0.850	3.111	0.168
4.888	0.772	3.094	0.230
4.830	0.869	3.016	0.361
4.847	0.820	2.871	0.523
4.649	1.112	1.818	1.259

T (K)	TmY		EcY	
	β_3-strand (%)			
	μ	σ	μ	σ
302	4.218	0.147	4.076	0.536
328	4.213	0.158	3.879	0.152
374	4.190	0.230	3.723	0.368
400	4.179	0.259	3.413	0.530
450	4.095	0.350	1.751	1.375

TmY		EcY	
β_4-strand (%)			
μ	σ	μ	σ
4.316	0.298	3.881	0.142
4.267	0.269	3.877	0.153
4.357	0.483	3.840	0.239
4.256	0.434	3.558	0.556
4.186	0.685	0.950	1.410

T (K)	TmY		EcY	
	β_5-strand (%)			
	μ	σ	μ	σ
302	3.316	0.481	2.674	0.403
328	3.225	0.512	2.803	0.418
374	2.996	0.776	2.700	0.492
400	3.073	0.760	2.584	0.700
450	2.657	1.130	0.594	1.026

12. Trends of each β-strand of the TmY and EcY proteins from three MD simulation replicas at the five analyzed temperatures.

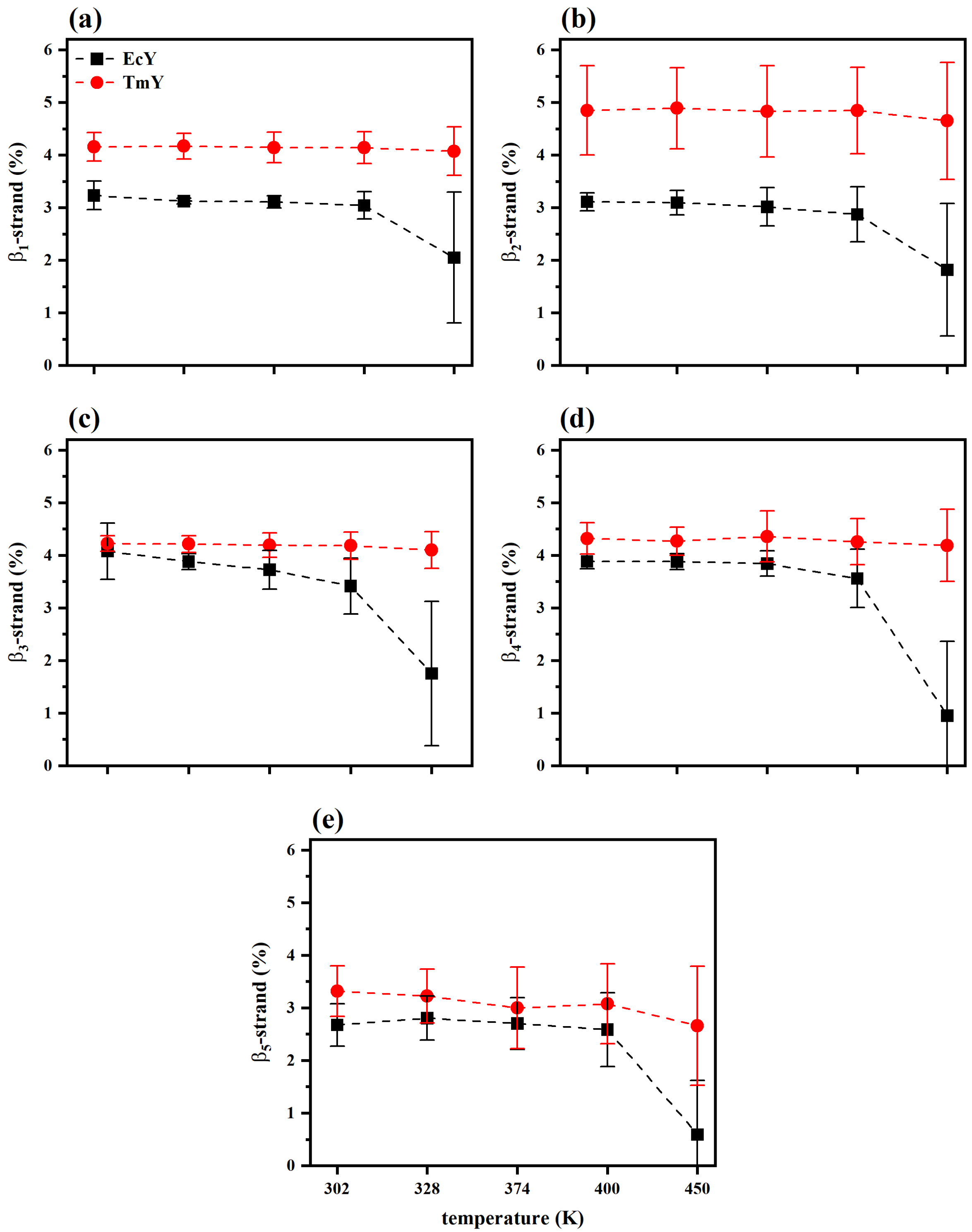

Figure S9. Average percentages and standard deviation values of (a) β_1-, (b) β_2-, (c) β_3-, (d) β_4-, (e) β_5 strands. Symbols represent the mean values, and error bars indicate the standard deviations. Dashed lines are shown as guides for the eye.

Description of β-strand percentages

As stated in the text of the manuscript, the behavior of β-strands differs from that of α-helices in the TmY protein. While α-helices progressively lose structure as the temperature increases (see Table S4), the β-strands remain almost unchanged (see Table S6), indicating a higher degree of thermal stability. In contrast, the β-strands of the EcY protein exhibit a similar trend to its α-helices, losing structure under the effect of temperature. Specifically, the TmY and EcY proteins lose on average 1.20 and 9.81% of their β-strand content, respectively, from 302 to 450 K. This observation is further supported by the boxplots (see Figure 7b), where the interquartile range IQR of EcY increases meaningfully at 450 K, spanning values between approximately 3.1 and 10.1%. In other words, half of the β-strand content is distributed within this range, with a median close to 7.80%, whereas at 302 K, 50% of the values are dispersed around 16.4 and 17.1%. In contrast, the TmY protein maintains 50% of its β-strands distributed around 20.3 and 21.2% at 302 K, with a slight decrease at 450 K, where the IQR lies between 18.8 and 21.3%.

The individual analysis of each β-strand from 302 to 450 K reveals distinct trends between the two proteins. The TmY protein loses on average only 0.66% of the β_5-strand, whereas the other four β-strands show an average loss of less than 0.21%. Conversely, the EcY protein loses approximately 1.2% of β_1 and β_2, more than 2.0% of β_3 and β_5, and nearly 3.0% of β_4. indicating that the hydrophobic core of EcY almost completely loses its β-strands as temperature increases.

13. Secondary structure profiles

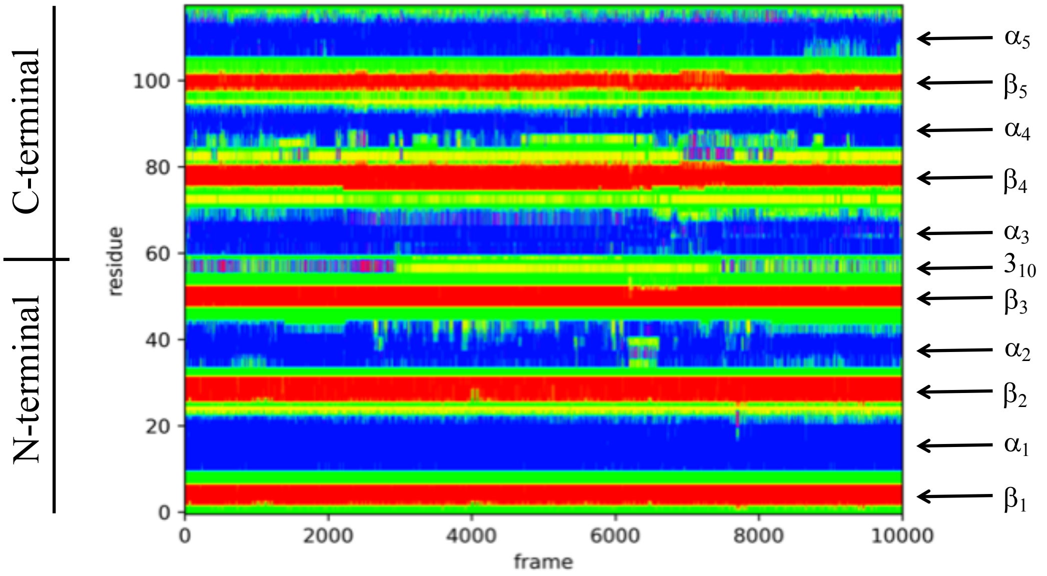

Figure S10. Secondary structure profile of the TmY protein achieved by DSSP algorithm. The screenshots correspond to the simulation 3 at 302 K. The color code of each structural pattern is: α-helix (blue), 3_{10}-helix (pink), β-strand (red), random coil (green), and turn (yellow).

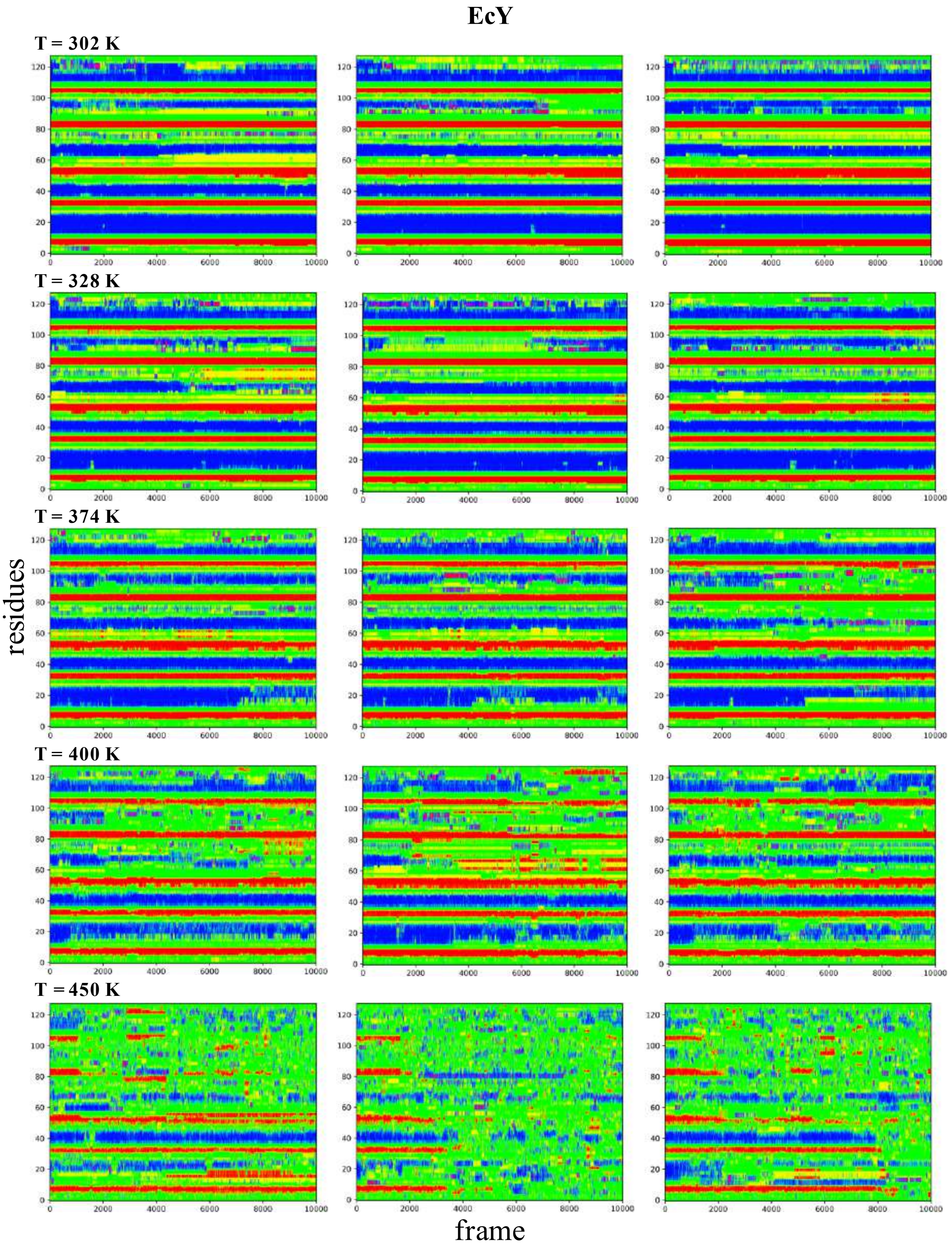

Figure S11. SS patterns of EcY protein from the three independent simulations at five temperatures. The color scheme follows that of Figure S9. The columns from left to right correspond to simulations 1, 2, and 3.

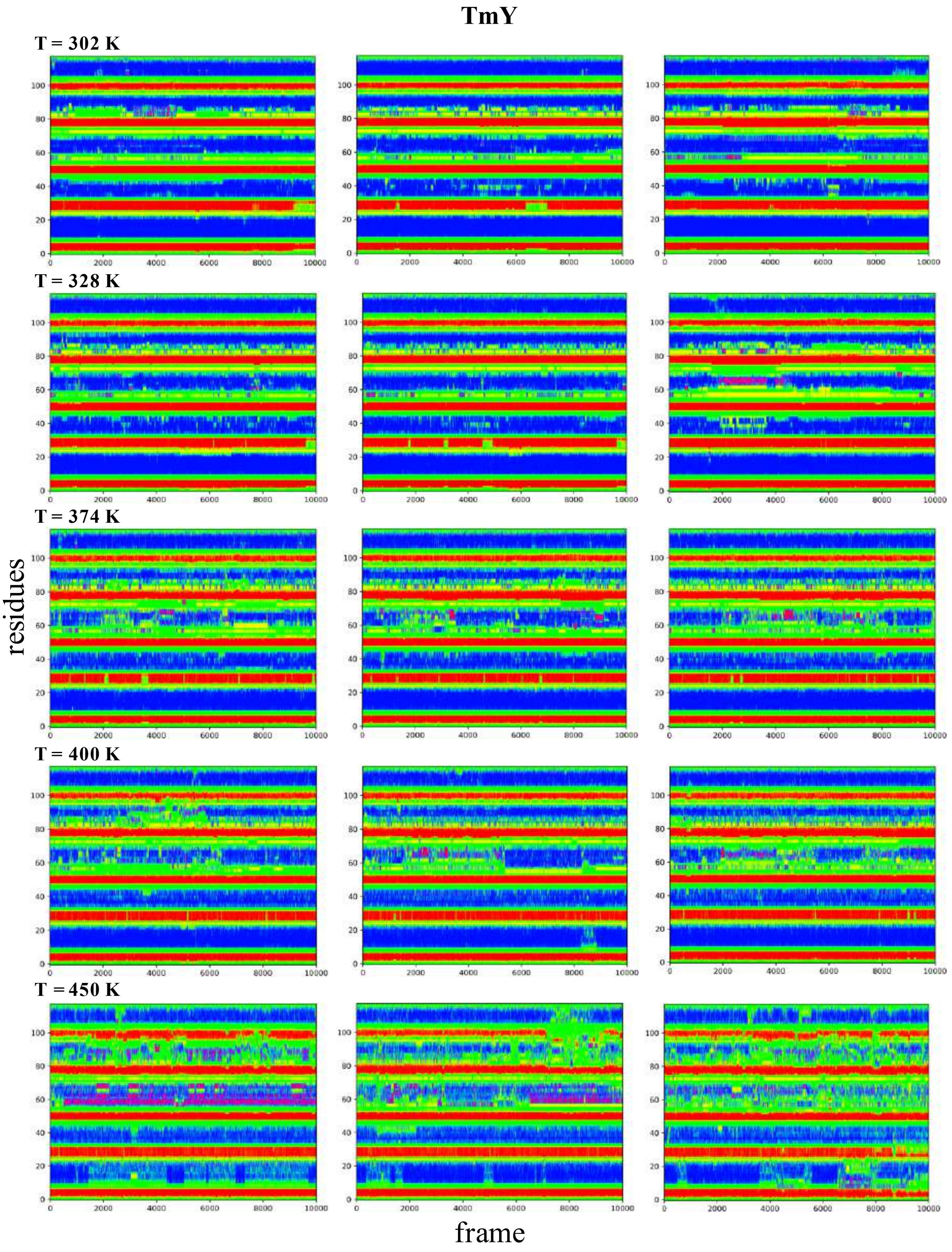

Figure S12. SS patterns of TmY protein from the three independent simulations at five temperatures. The color scheme follows that of Figure S9. The columns from left to right correspond to simulations 1, 2, and 3.

14. Root-mean-square fluctuation

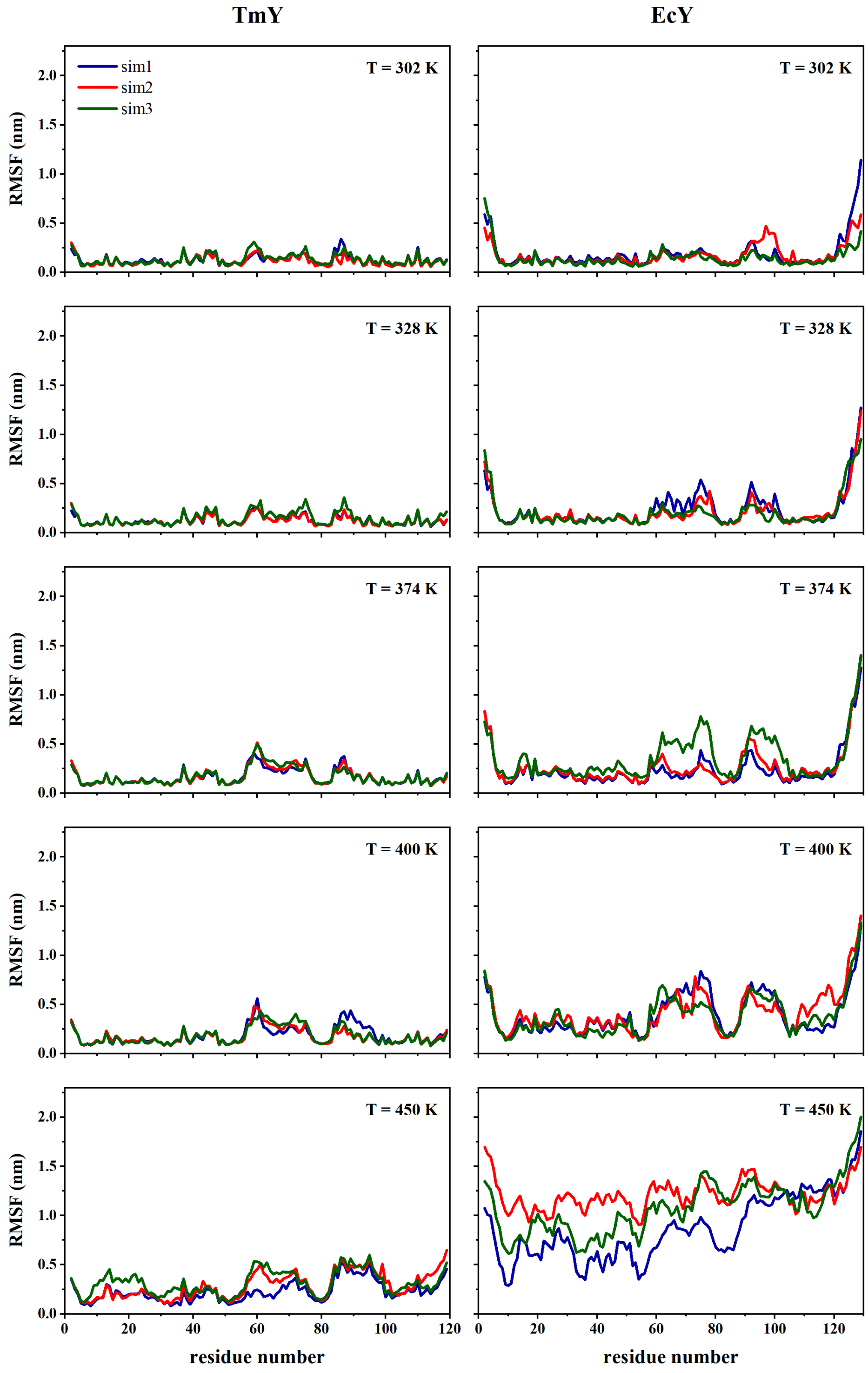

Figure S13. RMSF profiles of the individual replicas of TmY and EcY proteins at the five analyzed temperatures. The blue, red, and green lines indicate the simulations or replicas 1, 2, and 3, respectively.

Molecular interaction analyses

15. **Tables:** Statistical descriptors of the HBpp and HBps for the TmY and EcY proteins obtained from three MD simulation replicas at the five analyzed temperatures; μ = mean, σ = standard deviation, and m = median.

Table S8. Number of HBpp.

**T (K)**	**TmY**			**EcY**		
	HBpp					
	μ	σ	m	μ	σ	m
302	90.825	± 5.320	91.000	85.572	± 5.298	86.000
328	90.778	± 5.355	91.000	84.112	± 5.790	84.000
374	87.160	± 5.819	87.000	80.978	± 5.764	81.000
400	86.093	± 6.104	86.000	76.715	± 6.522	77.000
450	79.896	± 7.079	80.000	69.391	± 7.763	69.000

Table S9. Number of HBps.

**T (K)**	**TmY**			**EcY**		
	HBps					
	μ	σ	m	μ	σ	m
302	240.103	± 9.552	240.000	292.552	± 10.457	292.000
328	230.503	± 9.663	230.000	282.833	± 10.858	283.000
374	215.777	± 10.500	216.000	266.079	± 12.138	266.000
400	206.786	± 10.721	207.000	262.864	± 13.308	263.000
450	195.277	± 12.553	195.000	250.651	± 15.869	250.000

16. Multiple sequence alignment

```
                 10        20        30        40        50
EcY      MADKELKFLVVDDFSTMRRIVRNLLKELGFNNVEEAEDGVDALNKLQAGGYGFVISDWNM   60
StY      MADKELKFLVVDDFSTMRRIVRNLLKELGFNNVEEAEDGVDALNKLQAGGFGFIISDWNM   60
RmY      ---MKKRVLTVDDSRTIRNMLLVTLNNAGFETI-QAEDGVEGLEKLDTANPDVIVTDINM   56
HsY      ---MAKQVLLVDDSEFMRNLLREIL-EEEFEIADEAENGVEAVEMYKEYDPDLVMMDIVM   56
TmY      ---MGKRVLIVDDAAFMRMMLKDIITKAGYEVAGEATNGREAVEKYKELKPDIVTMDITM   57
BsY      ---MAHRILIVDDAAFMRMMIKDILVKNGFEVVAEAENGAQAVEKYKEHSPDLVTMDITM   57
               :.* ***   :* ::   : :  ::   :* :* :.::  .    ..:  *  *

                 70        80        90       100       110
EcY      PNMDGLELLKTIRADGAMSALPVLMVTAEAKKENIIAAAQAGASGYVVKPFTAATLEEKL   120
StY      PNMDGLELLKTIRADSAMSALPVLMVTAEAKKENIIAAAQAGASGYVVKPFTAATLEEKL   120
RmY      PRLDGFGFIEGVRKNDRYRAVPILVLTTESDAEKKNRARQAGATGWIVKPFDPTKLIDAI   116
HsY      PIRDGIEATSEIKEFDAGA--HIIMCTSIGQEEKMKKAVKAGADGYITKPFQKPSVMDAI   114
TmY      PEMNGIDAIKEIMKIDPNA--KIIVCSAMGQQAMVIEAIKAGAKDFIVKPFQPSRVVEAL   115
BsY      PEMDGITALKEIKQIDAQA--RIIMCSAMGQQSMVIDAIQAGAKDFIVKPFQADRVLEAI   115
         *  :*:   . :   .      ::: :: ..      * :*** .::.***    : : :

EcY      NKIFEKLGM      129
StY      NKIFEKLGM      129
RmY      ERVTA----      121
HsY      SDVLTA---      120
TmY      NKVSK----      120
BsY      NKTLN----      120
```

Figure S14. Multiple sequence alignment of CheY proteins from *Escherichia coli* (EcY), *Salmonella typhimurium* (StY), *Rhizobium meliloti* (RmY), *Halobacterium salinarum* (HsY), *Thermotoga maritima* (TmY), and *Bacillus subtilis* (BsY), generated using the Clustal Omega program. Conserved residues forming the Asp-Lys-Asp salt-bridge triad are highlighted by black boxes. Residue numbering is indicated every ten positions based on the EcY sequence to facilitate comparison among homologous positions.

17. Salt bridges in TmY

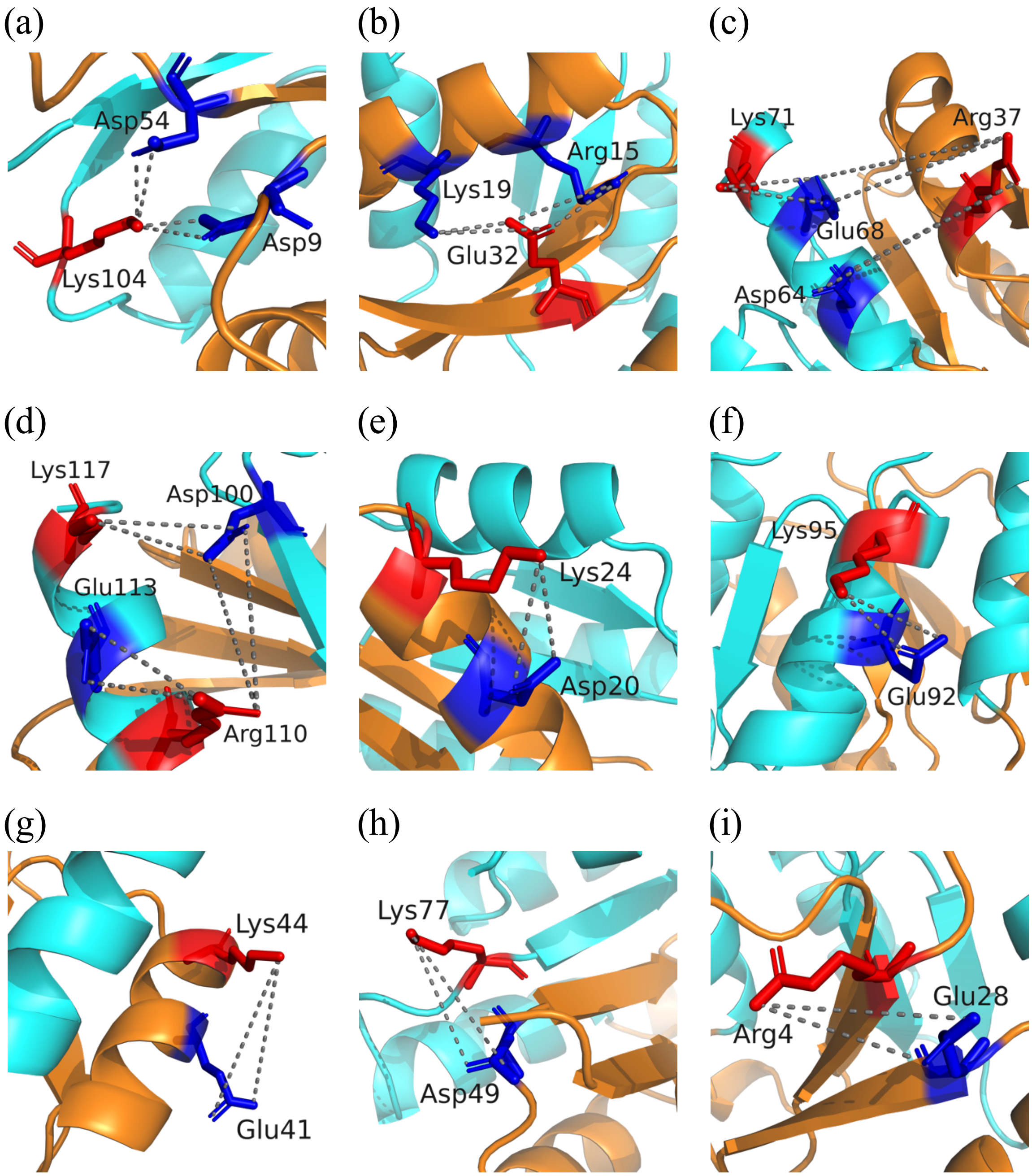

Figure S15. Spatial distribution of the fifteen salt bridges found in the TmY protein: (a) and (b) the Asp9···Lys104···Asp54 and Arg15···Glu32···Lys19 triads; (c) and (d) the Glu64···Arg37···Glu68···Lys71 and Glu113···Arg110···Asp100···Lys117 tetrads; (e), (f), (g), (h) and (i) the Asp20···Lys24, Glu92–Lys95, Glu41–Lys44, Asp49–Lys77, and Arg4–Glu28 dyads.
